# Metabolic glycoengineered exosome-A2M nanoplatform reprograms macrophage polarization and orchestrates bone regeneration in ONFH

**DOI:** 10.1038/s41420-025-02690-8

**Published:** 2025-11-07

**Authors:** Peng Chen, Ruisong Wang, Shanhong Fang

**Affiliations:** 1 Department of Sports Medicine, National Regional Medical Center, Binhai Campus of the First Affiliated Hospital, Fujian Medical University, Fuzhou, PR China; 2 Department of Orthopedic Surgery, The First Affiliated Hospital of Fujian Medical University, Fuzhou, PR China; 3 Fujian Orthopaedics Research Institute, Fuzhou, PR China; 4 Fujian Orthopedic Bone and Joint Disease and Sports Rehabilitation Clinical Medical Research Center, Fuzhou, PR China

**Keywords:** Inflammation, Immunopathogenesis

## Abstract

Osteonecrosis of the femoral head (ONFH), driven by glucocorticoid-induced M1 macrophage polarization and disrupted inflammatory homeostasis, poses a critical challenge in orthopedics. Here, we engineered adipose-derived mesenchymal stem cell exosomes (ADMSC-Exos) via metabolic glycoengineering (MGE) to deliver α2-macroglobulin (A2M), generating DS-exo@A2M. This nanoconstruct synergistically suppressed M1 polarization ( ↓ TNF-α, ↓IL-6) and promoted M2 polarization (↑CD206, ↑Arg-1) in M1 macrophages through IL-4 signaling activation, evidenced by transcriptomic/proteomic profiling and shRNA-mediated IL-4 knockdown. DS-exo@A2M further enhanced osteogenic differentiation of bone marrow-derived mesenchymal stem cells (BMSCs) by upregulating RUNX2, ALP, and OCN. In a rat ONFH model, DS-exo@A2M restored trabecular architecture ( ↑ BV/TV, ↓Tb.Sp) and reduced bone marrow edema. Mechanistically, IL-4 silencing abolished DS-exo@A2M-mediated macrophage reprogramming and osteogenesis, confirming pathway specificity. This study establishes a precision nanotherapeutic strategy for ONFH by integrating exosome engineering, immunomodulation and biosafety assessment, offering a translational framework for treating inflammation-associated bone disorders.

## Introduction

Osteonecrosis of the femoral head (ONFH) is a severe orthopedic disease characterized by interruption of blood supply to the femoral head, leading to cellular death and structural damage of bone [1–3]. The etiology of ONFH is complex and diverse, with common causes including trauma, prolonged use of glucocorticoids, excessive alcohol consumption, hematological disorders, and underlying genetic factors [2, 4, 5].

Macrophages play critical roles in immune regulation and tissue repair [6–8], existing as pro-inflammatory M1 (secreting TNF-α/IL-6) or anti-inflammatory M2 (secreting IL-10/TGF-β) phenotypes [9–11]. In glucocorticoid-induced ONFH, M1 dominance disrupts inflammatory homeostasis and exacerbates bone necrosis [12–14], while M2 reprogramming via cytokine modulation and gene silencing effectively suppresses inflammation and promotes osteogenesis [15–20], positioning macrophage phenotypic control as a promising therapeutic strategy [21, 22].

Exosomes are 30–150 nm nanovesicles mediating intercellular communication via proteins, nucleic acids, and lipids [23, 24], with superior biocompatibility, low immunogenicity, and barrier-crossing capabilities compared to traditional drug delivery systems [25, 26]. This study employs metabolic glycoengineering (MGE) to functionalize adipose-derived mesenchymal stem cell (ADMSC)-derived exosomes and loads them with α2-macroglobulin (A2M) via electroporation, creating DS-exo@A2M to enhance targeting specificity and therapeutic efficacy for disease treatment.

A2M exerts anti-inflammatory and immune-regulatory effects by modulating cytokine networks, suppressing inflammation, and promoting M2 macrophage polarization in orthopedic diseases [27–32], while IL-4 activates M2-specific pathways (CD206/Arg-1 expression) through receptor binding [11, 33–36]. Their synergistic combination may enhance inflammatory microenvironment remodeling in ONFH, improving bone repair through coordinated macrophage reprogramming [37].

This study develops exosome-mimetic scaffolds (DS-exo@A2M) to target A2M delivery, regulate macrophage polarization toward M2 phenotypes, remodel inflammatory microenvironments, and enhance bone repair in glucocorticoid-induced ONFH, employing NTA, TEM, Western blot, and multi-omics approaches (RNA-seq, proteomics) to elucidate molecular mechanisms of M1/M2 reprogramming. Preclinical validation in rat models confirms therapeutic efficacy through bone structural recovery and macrophage reprogramming, establishing DS-exo@A2M as a non-surgical exosome-based strategy with translational potential for ONFH treatment while advancing exosome engineering in orthopedic applications.

## Results

### Preparation and characterization of engineered exosomes loaded with A2M

**Fig. 1:**
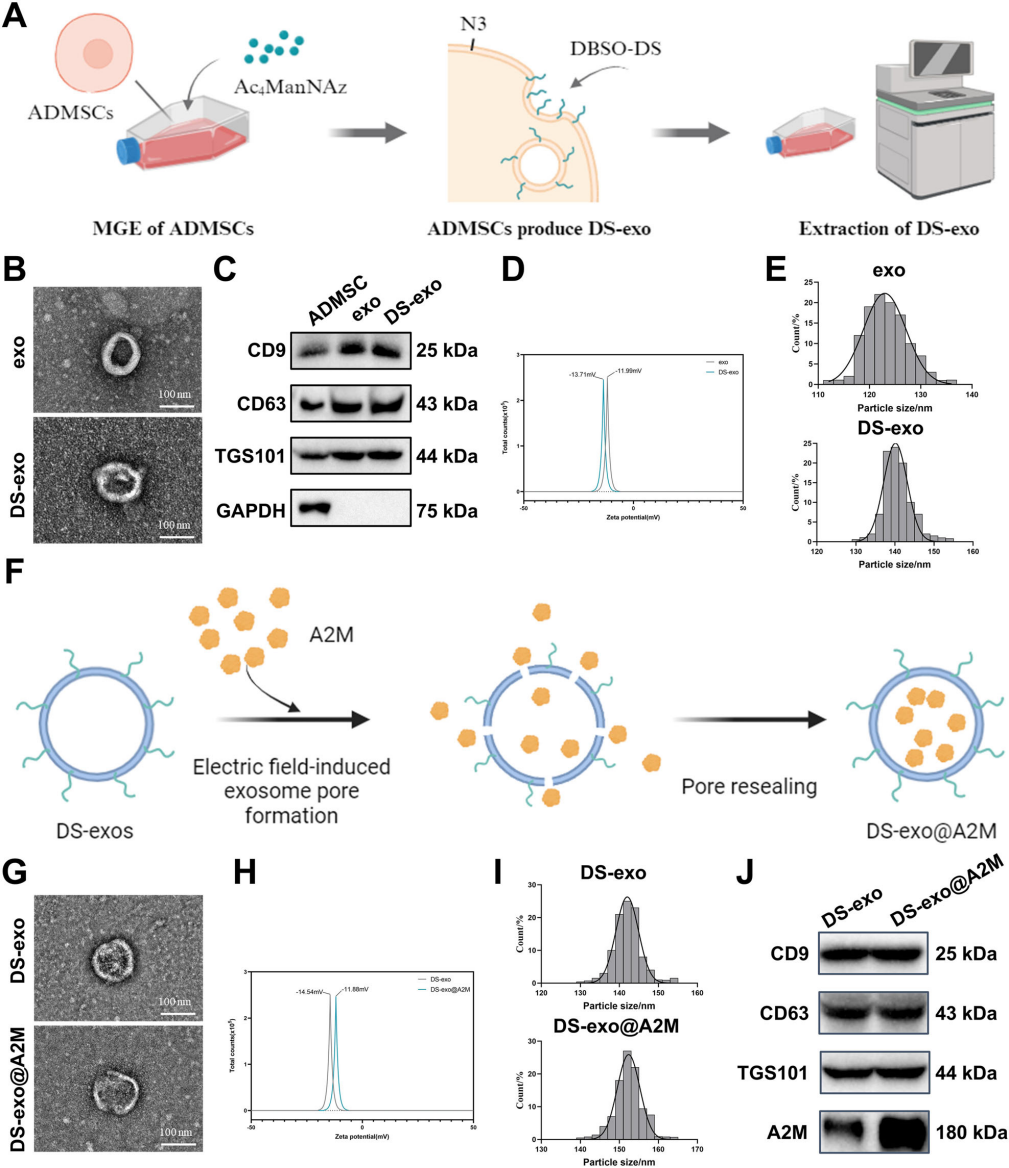
Engineering and Characterization of A2M-Loaded Exosomes. **A** Schematic illustration of the preparation of engineered exosomes; **B** TEM images of DS-exo and exo (scale = 100 nm); **C** Western Blot analysis of exosome markers; **D** Zeta potential analysis of DS-exo and exo using nanoparticle tracking; **E** Particle size distribution of DS-exo and exo analyzed by nanoparticle tracking; **F** Simplified process diagram of exosomes loaded with A2M; **G** TEM images of DS-exo and DS-exo@A2M (scale = 100 nm); **H** Zeta potential analysis of DS-exo and DS-exo@A2M using nanoparticle tracking; **I** Particle size distribution of DS-exo and DS-exo@A2M analyzed by nanoparticle tracking; **J** Western Blot detection of exosome markers and A2M.

Initially, we synthesized DBCO-conjugated cy5.5-labeled DS using click chemistry and attached it to ADMSCs’ surface containing the targeting fragment for scavenger receptor class A (SR-A) expressed on macrophages. The synthesized DBCO-DS displayed characteristic peaks of DS hydroxyl groups (-OH) (Fig. S1A-C). This process allowed us to obtain DS-exo, targeting macrophages (Fig. 1A). The biogenesis of exosomes is associated with endosome intraluminal vesicle formation, during which exosomal surface proteins like CD63 are integrated. To observe the integration of DS into exosomes, we treated ADMSCs expressing CD63-GFP with cy5.5-labeled DS. Confocal microscopy confirmed the colocalization of cy5.5-DS and CD63-GFP in cells after surface modification of ADMSCs through MGE-mediated click chemistry (Fig. S1D), demonstrating successful integration of DS into exosomes. TEM analysis of extracted exosomes revealed that DS-exo shared a similar circular or elliptical membrane-bound vesicle morphology as exosomes, with consistent size and intact structure (Fig. 1B). Western Blot identification of exosomal markers showed a significant increase in CD63, CD9, and TGS101 expression in exosomes compared to cell lysates, while Calnexin was barely expressed. DS-exo expressed exosomal markers similar to exosomes (Fig. 1C). NanoSight analysis indicated that both exosomes and DS-exo carried a negative charge (Fig. 1D), with DS-exo exhibiting slightly larger particle size (141.7 ± 8.1 nm) compared to exosomes (123.2 ± 7.3 nm) due to the presence of the DS shell (Fig. 1E). These results indicate successful extraction of well-dispersed DS-exo particles, where surface engineering through MGE-mediated click chemistry did not significantly affect the physical and chemical properties of exosomes.

Subsequently, A2M (approximately 180 kDa) was isolated and extracted from rat plasma (Fig. S1E confirms the extraction of A2M). The A2M was loaded into DS-exo using electroporation technique, designated as DS-exo@A2M (Fig. 1F). As depicted in Fig. 1G, the morphology of DS-exo@A2M appeared as circular or elliptical membranous vesicles, displaying consistent size and intact structure. NTA revealed that the average diameter of DS-exo@A2M was 150.20 ± 18.1 nm, with a Zeta potential of -11.88 mV. A slight increase in diameter and Zeta potential was observed for DS-exo@A2M compared to respective exosomes, indicating the integrity of DS-exo@A2M (Fig. 1H-I). Western Blot analysis demonstrated that the expression levels of CD63, CD9, and TSG101 in DS-exo@A2M were nearly identical to their respective exosomes, with a higher expression of A2M (Fig. 1J). These findings confirm the successful construction of DS-exo@A2M.

### DS-exo@A2M promotes macrophage reprogramming and BMSC osteogenic differentiation in ONFH

To verify the impact of DS-exo@A2M, prepared by us, on macrophage polarization in the ONFH environment, rat bone marrow-derived macrophages were induced with LPS to generate M1-type macrophages, and their polarization was confirmed by detecting the M1 marker proteins CD86 and CD80 (Fig. S2A).

**Fig. 2:**
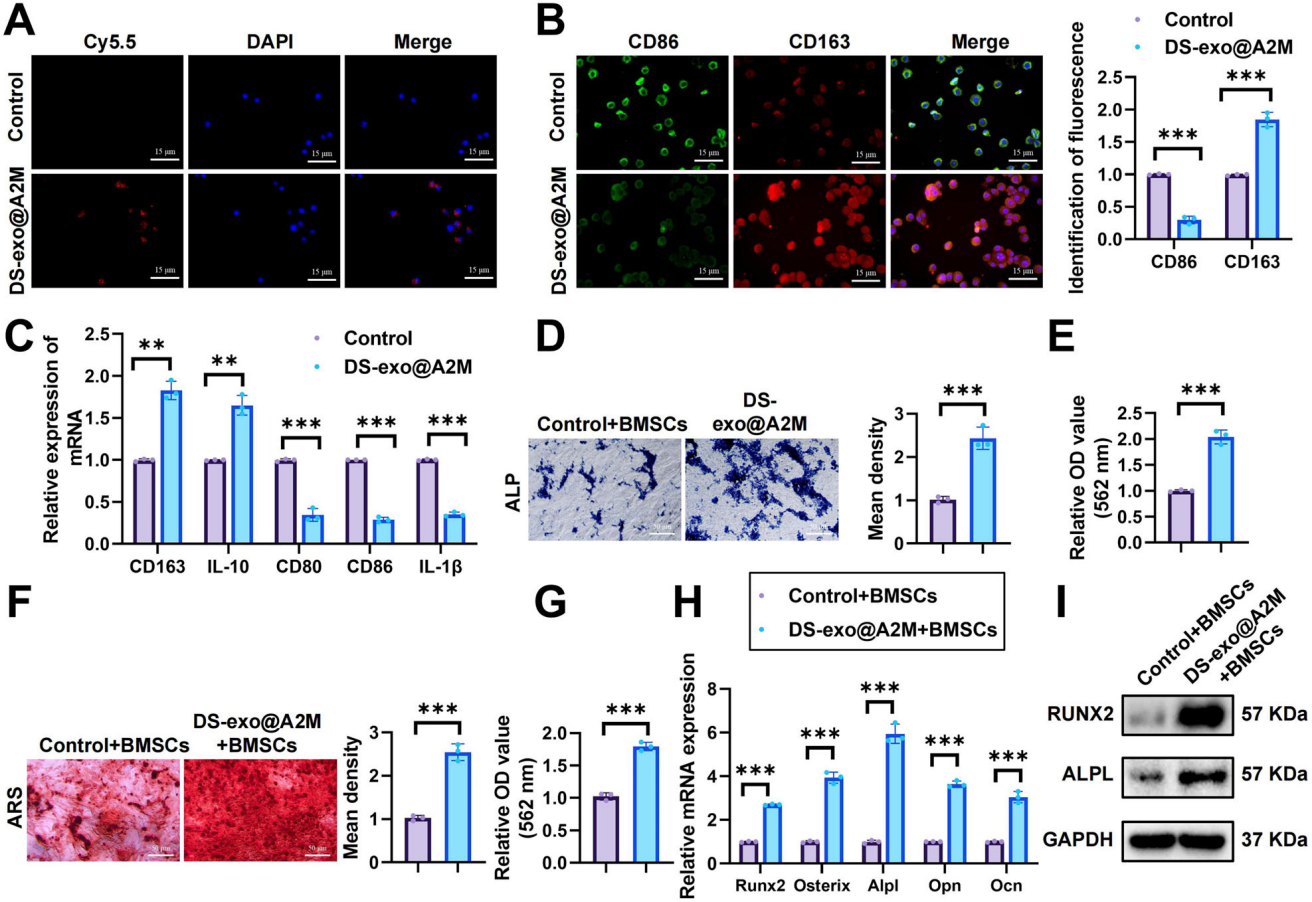
DS-exo@A2M Enhances Macrophage Reprogramming and BMSCs Osteogenic Differentiation. **A** Detection of macrophage uptake of DS-exo@A2M (scale bar = 15 μm). **B** Immunofluorescence analysis of the impact of DS-exo@A2M on M1 cell polarization (scale bar = 15 μm). Fluorescence quantification was performed relative to the control group threshold. **C** RT-qPCR analysis of the effect of PEG-NCCs@CA on M1 cell polarization. **D** ALP staining to assess ALP activity in BMSCs of each group (scale bar = 50 μm) **E** Measurement of ALP activity using an ALP activity assay kit. **F** ARS staining to evaluate osteogenic differentiation of BMSCs in each group (scale bar = 50 μm). **G** Quantification of ARS using a spectrophotometer. **H** RT-qPCR analysis of protein expression levels of osteogenic differentiation markers Runx2, Osterix, Alpl, Opn, and Ocn in each group of BMSCs. **I** Western Blot analysis of protein expression levels of osteogenic differentiation markers Runx2 and Alpl in each group of BMSCs **p < 0.01, ***p < 0.001; all cell experiments were conducted in triplicate.

Initially, immunofluorescence was employed to investigate the uptake of Cy5.5-labeled DS-exo@A2M by macrophages. The results demonstrated the presence of red fluorescence in macrophages co-cultured with DS-exo@A2M, indicating successful uptake (Fig. 2A).

Furthermore, immunofluorescence analysis revealed that treatment of M1 cells with DS-exo@A2M led to a significant decrease in CD86 fluorescence, while the fluorescence of the M2 macrophage marker CD163 notably increased compared to the control group (Fig. 2B). RT-qPCR results corroborated these findings, showing downregulation of M1 markers CD80, CD86, IL-1β, and upregulation of M2 markers CD163, IL-10 (Fig. 2C).

Research suggests that macrophages play a crucial role in mediating inflammatory responses and supporting the regenerative function of bone marrow stromal cells [38, 39]. To determine whether the reprogramming of macrophages promoted by DS-exo@A2M affects the osteogenic differentiation of BMSCs, rat BMSCs were procured. Induction assays confirmed the multipotency of BMSCs towards adipogenic, osteogenic, and chondrogenic lineages (Fig. S2B-C). Flow cytometry detected positive expression of cell markers CD90 and CD44, while markers IgG, hematopoietic markers CD45 and CD34 were negative (Fig. S2D), affirming the differentiation potential of BMSCs.

Next, macrophages and BMSCs were seeded at a 1:1 ratio in the upper and lower chambers, respectively, of Falcon® Cell Culture Inserts (Corning, Corning, NY). This setup was used to investigate the impact of DS-exo@A2M-treated macrophages on the osteogenic differentiation of BMSCs. ALP and ARS staining on the 7th and 21st day of osteogenic induction revealed significantly enhanced staining in the DS-exo@A2M group compared to controls (Fig. 2D-G). RT-qPCR analyses of osteogenic markers Runx2, Osterix, Alpl, Opn, and Ocn showed elevated expression levels in DS-exo@A2M-treated BMSCs (Fig. 2H), which was validated by Western Blot analysis (Fig. 2I). These results indicate that DS-exo@A2M-treated macrophages notably promote BMSC osteogenic differentiation.

### DS-exo@A2M promotes macrophage reprogramming by IL-4

**Fig. 3:**
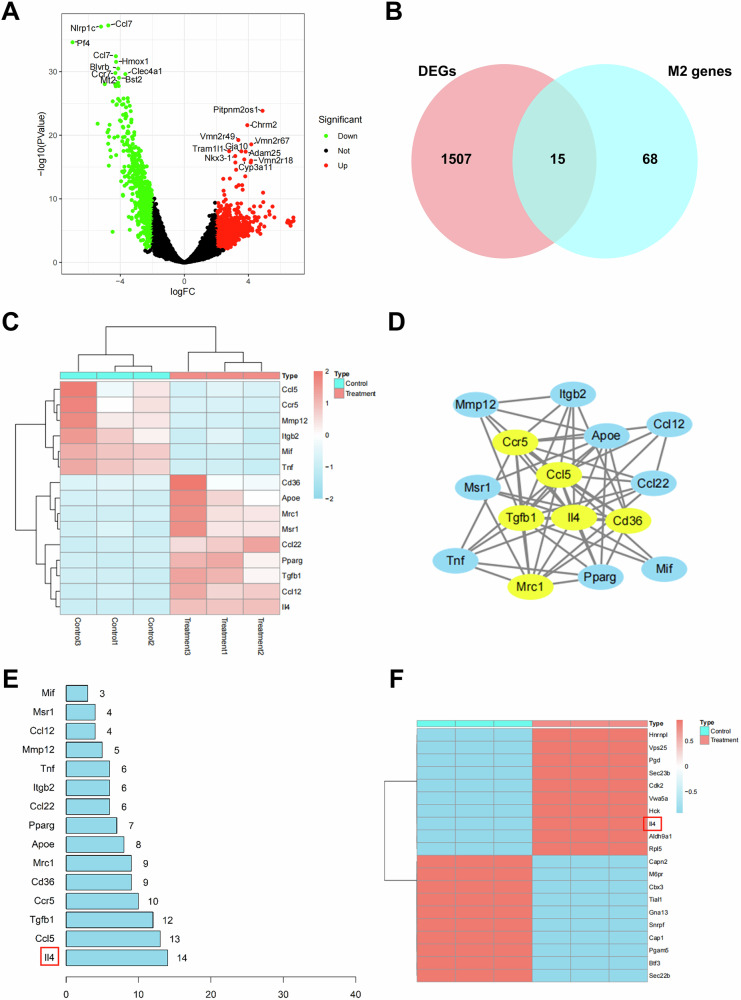
RNA-Seq and Proteomic Sequencing Data Analysis. **A** Volcano plot for differential expression analysis of RNA-Seq data, where red dots represent significantly upregulated genes, green dots represent significantly downregulated genes, and black dots represent genes with no differential expression, *p* <0.01, |log2FC| å 2; **B** Venn analysis of DEGs in relation to M2 macrophage-associated genes; **C** Heat map showing the gene expression of 15 M2-DEGs; **D** PPI network diagram for M2-DEGs analyzed using the STRING database, where yellow circles indicate core genes selected by Betweenness, Closeness, Degree, Eigenvector, LAC, and Network median criteria; **E** Network diagram displaying the final selection of 6 core genes after screening; **F** Protein interaction Degree values of M2-DEGs analyzed by the STRING database; **F** Heat map showing differential expression of proteomic sequencing data; *p* <0.01, |log2FC| å 2, each group with n = 3.

To further investigate the impact of DS-exo@A2M, we conducted RNA-seq on treated M1 macrophages. Differential gene expression analysis of RNA-seq data from Control group (n = 3) and Treatment group (n = 3) yielded 1517 DEGs, including 629 downregulated and 888 upregulated genes (Fig. 3A). Principal Component Analysis (PCA) revealed distinct separation of Control and Treatment samples along PC1 and PC2 axes, indicating significant differences in gene expression levels between the two groups (Fig. S3A). Functional enrichment analysis of DEGs through GO and KEGG pathways demonstrated enrichment in inflammatory-related terms such as leukocyte migration, cell chemotaxis, and cytoplasmic translation (Fig. S3B). GSEA revealed a significantly downregulated innate immune system pathway with a lower Normalized Enrichment Score (-4.758) post treatment with DS-exo@A2M (Fig. S3C-D). Venn analysis of the DEGs with the top 100 genes related to “M2 macrophage” in the GeneCard database identified 15 highly correlated DEGs with M2 macrophages (Fig. 3B) (termed M2-DEGs), of which 9 genes were upregulated and 6 genes were downregulated (Figs. 3C, S3E).

Subsequently, protein-protein interaction (PPI) analysis of the M2-DEGs was performed using the STRING database (https://cn.string-db.org/) with a medium confidence cutoff score of 0.400, resulting in a PPI network comprising 15 nodes and 58 edges (Fig. 3D). Importing the analysis into Cytoscape software and filtering based on topological analysis using metrics such as Betweenness, Closeness, Degree, Eigenvector, LAC, and Network Median, a core PPI network consisting of 6 proteins with 15 edges was obtained (Fig. 3D, Table S1). Among these, IL-4 ranked first in degree centrality (Fig. 3E), with its expression profile shown in (Fig. S3F). IL-4, a critical anti-inflammatory cytokine, promotes macrophage polarization towards the M2 phenotype, exerting anti-inflammatory and tissue repair effects [40].

Additionally, proteomic sequencing was conducted on Control group (n = 3) and Treatment group (n = 3) macrophages. PCA results and Loading plot indicated a clear separation between Control and Treatment groups (Fig. S4A-C). Orthogonal Partial Least Squares Discriminant Analysis (OPLS-DA) showed distinct separation of Control and Treatment groups in the OPLS-DA score plot, with R2Y = 0.999 > 0.8 in the permutation analysis, confirming the stability of the model (Fig. S4D-F). Subsequent differential protein expression analysis revealed a total of 68 differentially expressed proteins, including 45 upregulated and 23 downregulated proteins, with IL-4 among the upregulated proteins (Figs. 3F, S4G). GO and KEGG analysis results indicated enrichment of these differentially expressed proteins in pathways related to inflammatory responses, such as cellular response to biotic stimulus, molecule of bacterial origin, and LPS, highlighting the crucial role of DS-exo@A2M treatment in maintaining immune balance (Fig. S4H).

To investigate whether IL-4 acts as a downstream regulator of DS-exo@A2M-mediated macrophage reprogramming, we established IL-4-silenced macrophage stable transfectants (designated as sh-IL-4) (validated for better silencing efficiency with sh-IL-4-1 chosen for subsequent experiments, referred to as sh-IL-4) (Fig. S4I-J).

**Fig. 4:**
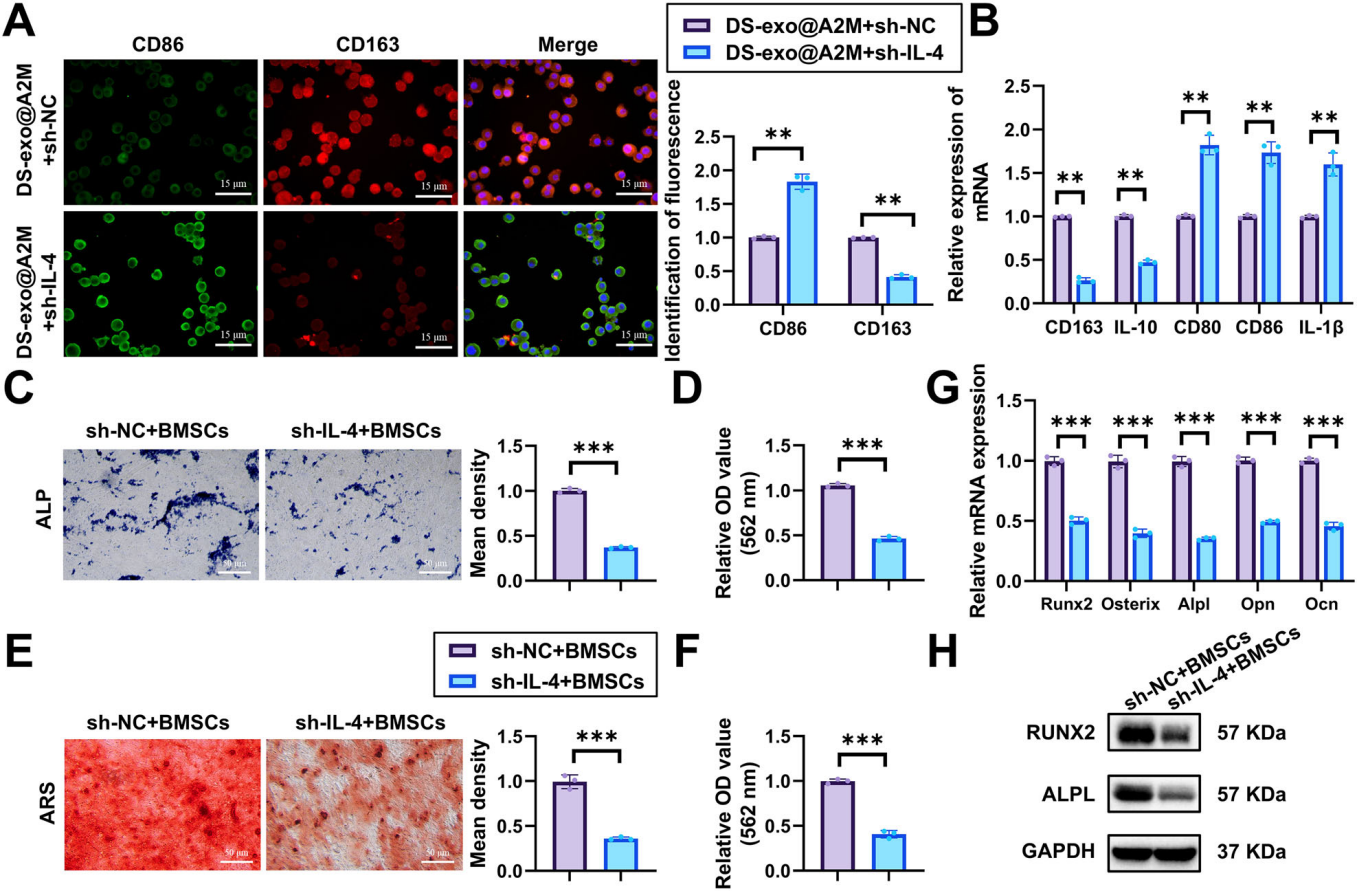
DS-exo@A2M promotes macrophage reprogramming via IL-4 stimulation. **A** Immunofluorescence analysis of the impact of silencing IL-4 on M1 cell polarization (scale bar = 15 μm); **B** RT-qPCR analysis of the effect of silencing IL-4 on M1 cell polarization; **C** ALP staining to assess ALP activity in different BMSCs groups (scale bar = 50 μm); **D** ALP activity measurement using an ALP activity assay kit; **E** ARS staining to evaluate osteogenic differentiation of BMSCs in different groups (scale bar = 50 μm); **F** Quantification of ARS using a spectrophotometer; **G** RT-qPCR analysis of the gene expression levels of osteogenic differentiation markers Runx2, Osterix, Alpl, Opn, and Ocn in different BMSCs groups; **H** Western Blot analysis of the protein expression levels of osteogenic differentiation markers Runx2 and Alpl in different BMSCs groups. Statistical significance is denoted as **p < 0.01, ***p < 0.001; all cell experiments were performed in triplicate.

Immunofluorescence results revealed that compared to DS-exo@A2M+sh-NC, cells treated with DS-exo@A2M+sh-IL-4 exhibited significantly increased CD86 fluorescence and decreased CD163 fluorescence, a hallmark protein of M2 macrophages (Fig. 4A). Consistent with the immunofluorescence findings, RT-qPCR results demonstrated upregulation of M1 cell markers CD80, CD86, and IL-1β, while downregulation of M2 cell markers CD163 and IL-10 (Fig. 4B). These results indicate that DS-exo@A2M influences macrophage reprogramming through IL-4.

Subsequently, we co-cultured IL-4-silenced macrophages treated with DS-exo@A2M with BMSCs. On the 7th and 21st day of inducing osteogenic differentiation in BMSCs, ALP and ARS staining were performed, revealing a significant reduction in ALP and ARS staining after IL-4 silencing compared to the control group (Fig. 4C-F). RT-qPCR analysis of osteogenic markers Runx2, Osterix, Alpl, Opn, and Ocn in BMSCs showed a significant decrease in the expression levels in the sh-IL-4-BMSCs group compared to sh-NC-BMSCs group (Fig. 4G). Western Blot results were consistent with these findings (Fig. 4H). These findings suggest that DS-exo@A2M influences BMSC osteogenic differentiation by modulating IL-4 to enhance macrophage reprogramming.

### DS-exo@A2M promotes reprogramming of macrophages and bone tissue repair in a rat model of ONFH

**Fig. 5:**
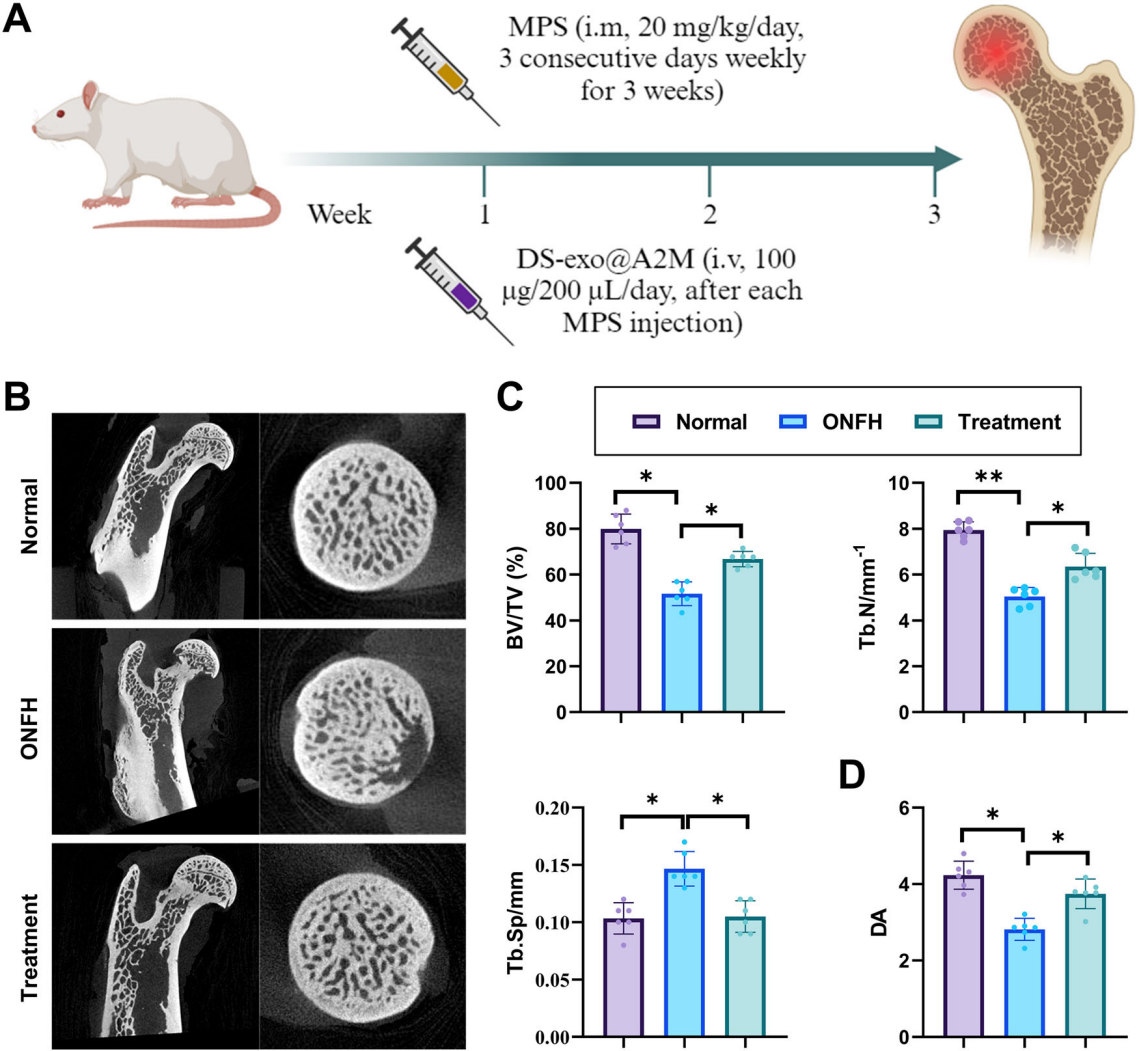
DS-exo@A2M promotes bone tissue repair in rats with ONFH. **A** Schematic illustration of DS-exo@A2M treatment in rats with ONFH; **B** Representative Micro-CT scan images of rat femoral heads in each group; **C** Quantitative bone density and related indices of bone quality in femoral heads: BV/TV, Tb.N, and Tb.Sp; **D** Quantitative directional structural indices of bone tissue, DA; **p < 0.05, ***p < 0.01; n = 6 in each group.

To further investigate the therapeutic effect of DS-exo@A2M on ONFH in vivo, we established a rat model of ONFH induced by glucocorticoids (Fig. 5A), with rats randomly assigned to the Normal group, ONFH group, and Treatment group. Flow cytometry analysis confirmed that DS-exo@A2M could also be taken up by macrophages in vivo (Fig. S5A).

Initially, Micro-CT was utilized to examine the trabecular structure and overall integrity of bone in the femoral heads of rats in each group. As shown in Fig. 5B, compared to the Normal group, the ONFH model group exhibited significant alterations in bone microstructure, characterized by sparse and irregular trabecular structures and overall structural disarray, reflecting features of osteoporosis and structural damage. Following DS-exo@A2M treatment, the trabecular structures showed restoration, and the overall structure appeared more intact. Furthermore, quantification was performed on bone density and quality-related parameters BV/TV, Tb. N, Tb. Sp, and the directional indicator of bone tissue structure, DA. The results revealed that BV/TV and Tb.N were significantly lower, while Tb.Sp was significantly higher in the ONFH model group compared to the Normal group, indicating bone loss in the femoral heads of rats with ONFH. Post DS-exo@A2M treatment, BV/TV and Tb.N values increased significantly, while Tb.Sp decreased markedly, suggesting that DS-exo@A2M treatment prevented bone loss (Fig. 5C). Additionally, the decrease in DA indicated a weakened directional alignment of trabeculae, which was restored by DS-exo@A2M treatment, enhancing the overall mechanical strength and stability of bones (Fig. 5D).

**Fig. 6:**
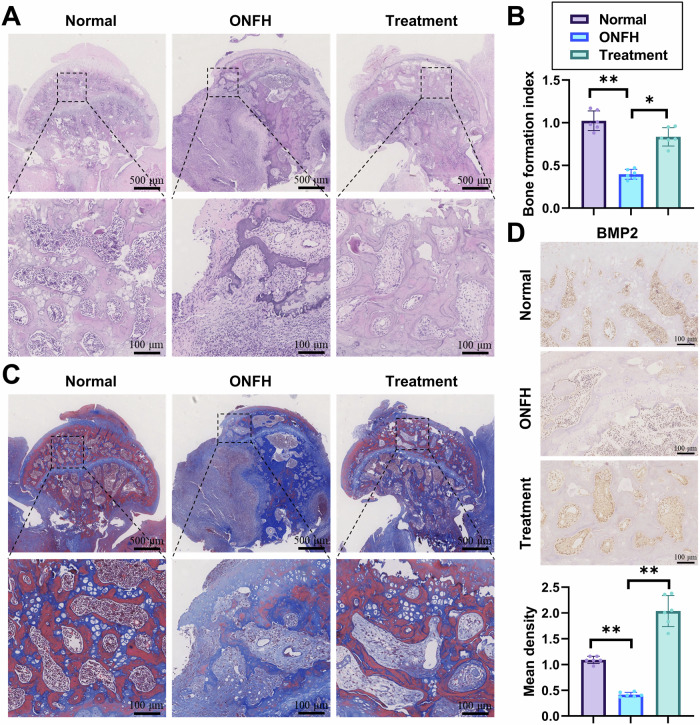
DS-exo@A2M Promotes Bone Tissue Repair in Rat Osteonecrosis. **A** Histological analysis of rat femoral heads in each group (scar = 100 μm); **B** Semi-quantitative analysis of new bone formation; **C** Masson’s trichrome staining of rat femoral heads in each group (scar = 100 μm); **D** Immunohistochemical staining for BMP2 in rat femoral heads in each group (scar = 100 μm). (*p < 0.05, **p < 0.01); n = 6 in each group.

H&E staining revealed that, compared to the Normal group, the ONFH group exhibited defective areas in the femoral heads covered by bone collagen, whereas the Treatment group showed evidence of new bone tissue (Fig. 6A). Further assessment of bone repair was conducted using the bone formation index, indicating severe defects and minimal bone regeneration in the ONFH group, while the Treatment group displayed increased new bone formation (Fig. 6B). Results from Masson’s staining displayed more mature collagen fibers in the Treatment group compared to the ONFH group (Fig. 6C). Immunohistochemistry staining exhibited high expression of BMP2 (a protein inducing osteogenic differentiation) in the Treatment group (Fig. 6D). These findings suggest that the femoral head defects, reduced bone tissue regeneration, lower collagen fibers, and decreased BMP2 expression observed in the ONFH group were reversed by DS-exo@A2M treatment.

**Fig. 7:**
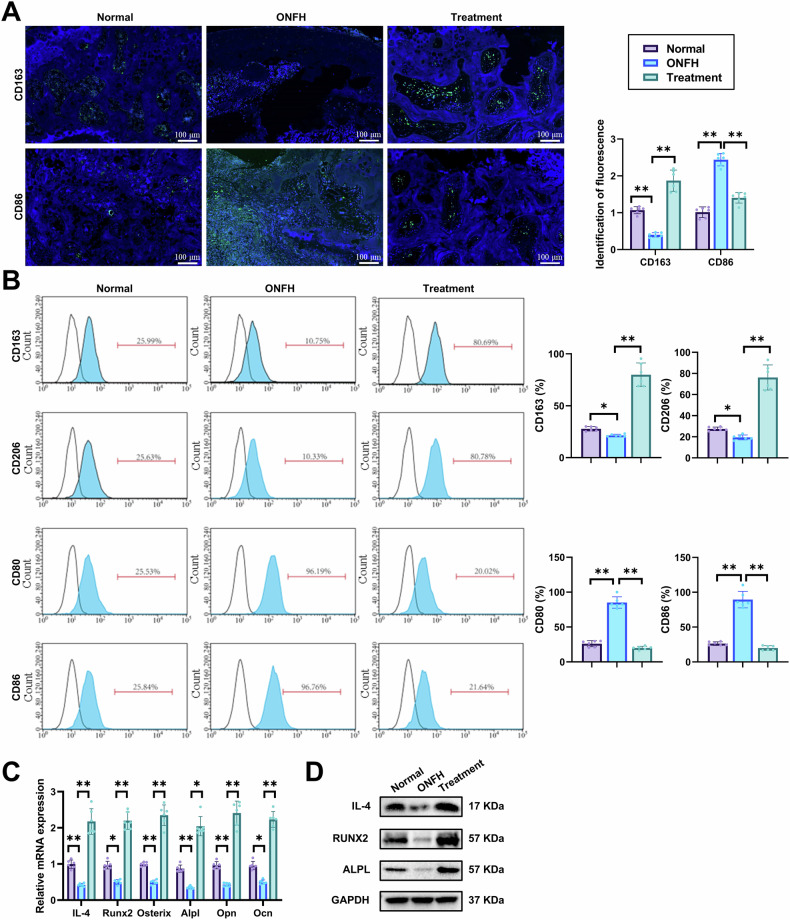
DS-exo@A2M promotes reprogramming of macrophages in the bone tissue of rats with ONFH. **A** Immunofluorescence detection of the polarization of bone tissue macrophages in rats with ONFH treated with DS-exo@A2M (scar = 100 μm); **B** Flow cytometry analysis of the polarization of bone tissue macrophages in rats with ONFH treated with DS-exo@A2M; **C** RT-qPCR assessment of the gene expression levels of osteogenic differentiation markers Runx2, Osterix, Alpl, Opn, Ocn, and IL-4 in the femoral heads of rats in each group; **D** Western Blot analysis of the protein expression levels of osteogenic differentiation markers Runx2, Alpl, and IL-4 in the femoral heads of rats in each group; *p < 0.05, **p < 0.01; n = 6 per group.

In order to further investigate the impact of DS-exo@A2M treatment on macrophages in vivo, we utilized immunofluorescence staining to analyze the expression of specific phenotypic markers for M1 macrophages (CD86) and M2 macrophages (CD163) in the femoral heads of rats in each group. The results revealed that, compared to the Normal group, the ONFH group exhibited significantly enhanced CD86 fluorescence, which was notably diminished following DS-exo@A2M treatment, accompanied by increased CD163 fluorescence intensity (Fig. 7A). Flow cytometry results were consistent with immunofluorescence findings (Fig. 7B). Additionally, RT-qPCR and Western Blot experiments demonstrated that in the femoral heads of rats in the ONFH group, the expression levels of IL-4 and osteogenic differentiation markers were downregulated, whereas their expression was significantly upregulated post DS-exo@A2M treatment (Fig. 7C, D). These findings suggest that DS-exo@A2M treatment in vivo promotes M2 macrophage polarization and osteogenic differentiation.

Furthermore, we conducted a safety assessment of DS-exo@A2M in vivo. Results indicated no apparent damage in the hearts, livers, spleens, lungs, and kidneys of rats in any group based on H&E staining (Fig. S5B). Serum biochemical analyses revealed no significant differences in liver function markers (ALT, AST), heart function markers (CK, LDH), or kidney function markers (CREA, urea) (Fig. S5C-E).

## Discussion

DS-exo@A2M effectively modulates M1-to-M2 macrophage polarization and promotes osteogenic differentiation of BMSCs, thereby ameliorating bone structural damage and enhancing new bone formation in glucocorticoid-induced ONFH rats. These findings demonstrate its therapeutic potential for macrophage-mediated inflammatory microenvironment remodeling and bone repair in ONFH.

Macrophages are central to inflammatory regulation and tissue repair [41–43], but conventional drug/gene therapies [44] suffer from poor targeting and systemic toxicity [7, 45–47]. This study overcomes these limitations by engineering DS-exo@A2M – an exosome-mimetic scaffold delivering A2M to modulate M1/M2 polarization via IL-4 signaling [37] – combined with metabolic glycoengineering (MGE)-enhanced targeting, achieving superior bone repair efficacy in ONFH models with minimized off-target effects.

Exosomes possess inherent advantages in biocompatibility, low immunogenicity, and barrier-crossing capacity [48–50], which were enhanced in this study through metabolic glycoengineering (MGE)-modified surfaces and electroporation-based A2M loading. Engineered DS-exo@A2M outperforms traditional drug delivery systems by protecting A2M stability, enabling tissue-specific targeting to reduce off-target effects [48, 49], and improving therapeutic efficacy through synergistic M2 macrophage polarization modulation [37].

A2M exerts anti-inflammatory and immune-regulatory effects by modulating cytokine networks and protecting tissues [29, 30, 51–53], which were enhanced in this study through exosome-based delivery. Mechanistic studies revealed that DS-exo@A2M promotes M2 macrophage polarization via IL-4 signaling [31, 37], reshaping the inflammatory microenvironment to stimulate bone repair in ONFH. Compared to conventional anti-inflammatory drugs, A2M demonstrates superior specificity and efficacy in macrophage reprogramming, positioning it as a promising therapeutic candidate [37].

IL-4 plays a critical role in promoting M2 macrophage polarization [11, 33, 34], and this study demonstrated that DS-exo@A2M enhances its regulatory efficacy through synergistic IL-4 signaling, as evidenced by upregulated IL-4 expression and M2 polarization [11, 33, 34, 37]. Compared to monotherapy with IL-4 or A2M, DS-exo@A2M achieved superior therapeutic outcomes in macrophage reprogramming, validating the combinatorial advantage of exosome-mediated immune modulation [37].

DS-exo@A2M significantly ameliorates bone structural damage and promotes new bone formation in glucocorticoid-induced ONFH rats, as evidenced by Micro-CT, H&E, and Masson staining, while enhancing M2 macrophage polarization and suppressing inflammation via immunofluorescence and flow cytometry. Compared to conventional therapies, DS-exo@A2M demonstrates superior bone repair efficacy, specificity, and safety profile, positioning it as a promising novel treatment for ONFH.

In this study, we successfully developed engineered exosomes (DS-exo@A2M), which promote bone repair in osteonecrosis of the femoral head (ONFH) by regulating IL-4-mediated M2 macrophage polarization and reshaping the inflammatory microenvironment. This finding offers a promising non-surgical strategy for ONFH treatment and opens new avenues for the application of exosomes in orthopedic diseases.

Despite these encouraging results, several limitations remain. First, the sample size was relatively small, and further validation in larger-scale studies is necessary. Second, long-term efficacy and safety were not evaluated due to the absence of extended follow-up data. Future research should include expanded sample sizes and multicenter trials to confirm therapeutic potential.

Additionally, the study did not explore whether the exosome concentration used reached the uptake saturation point in macrophages or assess their retention time. Further experiments will be conducted to determine the optimal and biologically relevant dosing. Although DS-exo@A2M effectively promoted bone repair by modulating macrophage polarization, its direct impact on osteogenic differentiation remains unclear. Follow-up studies will investigate its interaction with BMSCs.

Moreover, our model involved DS-exo@A2M administration during ONFH induction; its therapeutic effects after ONFH establishment were not assessed. Future studies will address this to provide more clinically relevant evidence. Lastly, the potential applications of DS-exo@A2M in other orthopedic conditions warrant exploration to advance its clinical translation. With continued investigation, DS-exo@A2M may offer a novel and effective therapeutic option for patients with ONFH and other skeletal disorders.

## Conclusion

**Fig. 8:**
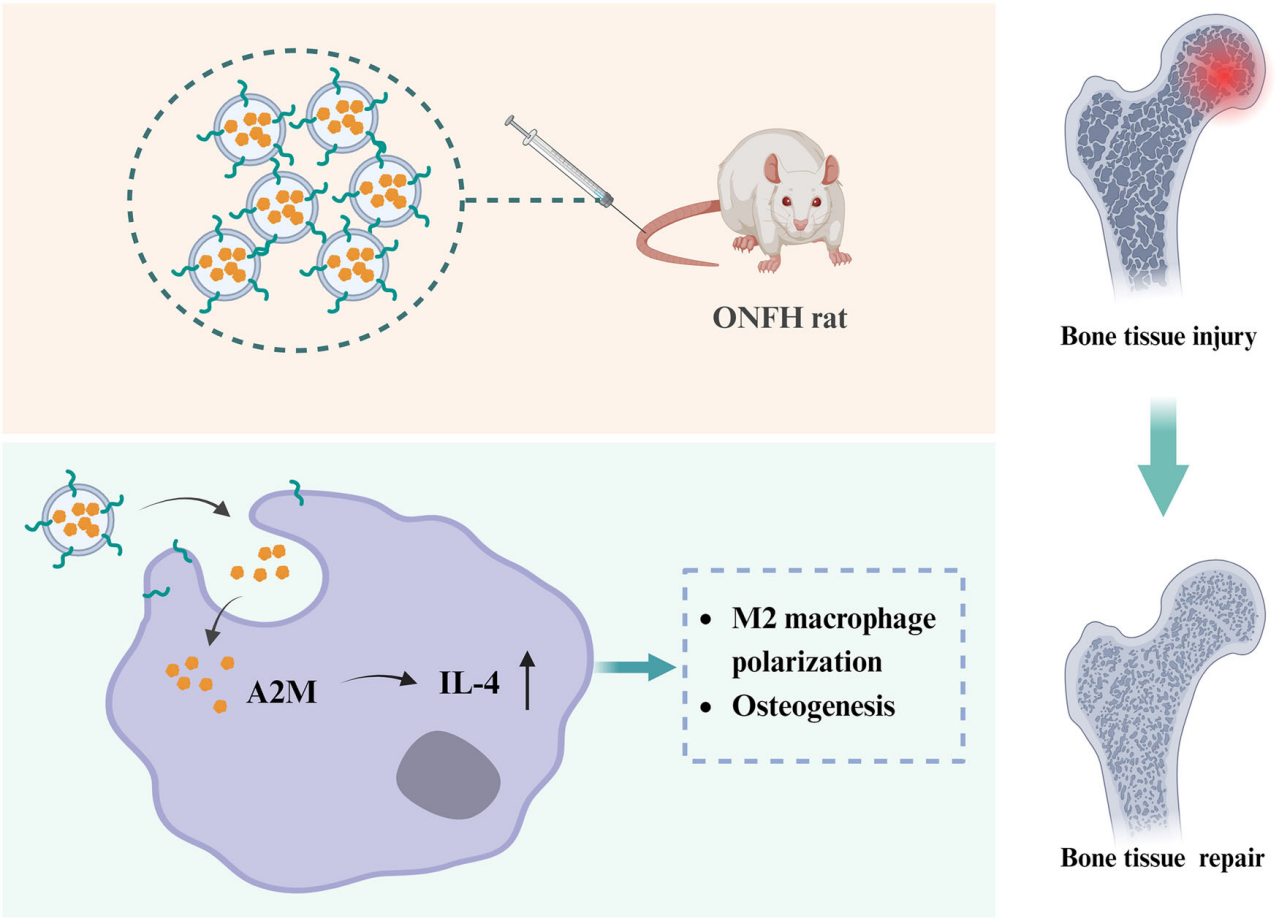
Engineered exosome biomimetic scaffold for targeted delivery of A2M promotes bone repair in avascular necrosis of the femoral head by regulating macrophage polarization to the M2 phenotype through IL-4 to remodel the inflammatory microenvironment.

This study developed an engineered exosome DS-exo@A2M to regulate IL-4-mediated polarization of macrophages towards M2 phenotype and reshape the inflammatory microenvironment of ONFH, leading to successful restoration of ONFH (Fig. 8). This innovative therapeutic strategy demonstrated significant efficacy in animal models, laying a crucial foundation for clinical translation.

Characterization through NTA, transmission electron microscopy, and Western Blot validation confirmed the successful preparation and functionality of DS-exo@A2M, showing its capability to effectively suppress M1 macrophage polarization and promote M2 polarization. In vivo experiments further supported its ability to ameliorate bone structural damage and enhance new bone formation in the corticosteroid-induced ONFH model.

This study confirms the potential of DS-exo@A2M in improving ONFH and promoting M2 macrophage polarization. However, the mechanism by which DS-exo@A2M enhances symptoms through promoting M2 polarization remains unresolved. Additionally, while observing the promotion of M2 polarization by DS-exo@A2M through the IL-4 pathway, further in-depth research is needed to elucidate how DS-exo@A2M molecularly regulates IL-4 to impact macrophage polarization and bone repair processes. Future investigations should delve into clarifying the detailed signaling pathways through which DS-exo@A2M facilitates macrophage polarization via IL-4 and how these pathways specifically influence the therapeutic process of ONFH. Exploring the feasibility of engineering exosomes that combine with other bioactive molecules may pave the way for novel approaches to treating a broader spectrum of bone disorders. Through multicenter, large-scale clinical trials, validating the clinical potential of these strategies will propel significant advancements in the field of bone tissue regenerative medicine.

## Materials and methods

This study outlines a systematic approach to engineer and validate an exosome-based nanotherapeutic platform for osteonecrosis therapy. Key methodologies include:

Exosome Engineering: Adipose-derived mesenchymal stem cell exosomes (ADMSC-Exos) were metabolically modified via surface conjugation of dibenzocyclooctyne-functionalized dextran sulfate (DBCO-DS) and subsequently loaded with α2-macroglobulin (A2M) using electroporation to generate the hybrid scaffold DS-exo@A2M.

Characterization: Exosome identity was confirmed through nanoparticle tracking analysis (NTA) to assess size distribution and zeta potential, transmission electron microscopy (TEM) for morphological evaluation, and Western blot analysis of exosomal markers (TSG101, CD9, CD63) and endoplasmic reticulum contaminant (Calnexin). Cellular uptake efficiency was quantified via confocal microscopy and flow cytometry following macrophage incubation with DS-exo@A2M.

In Vitro Models: Rat adipose-derived mesenchymal stem cells (ADMSCs; CP-R198, Procell) and bone marrow-derived mesenchymal stem cells (BMSCs; CP-R131, Procell) were cultured in α-MEM supplemented with 15% FBS and antibiotics. BMSCs were characterized by flow cytometry (CD44, CD90, CD45, CD34) and tri-lineage differentiation assays (osteogenic: ALP/Runx2/OCN; adipogenic: Oil Red O; chondrogenic: Alcian Blue). M1 macrophage polarization was induced in rat bone marrow-derived macrophages (CP-R141, Procell) using IFN-γ/LPS stimulation. Co-culture systems were established using Falcon cell culture inserts to investigate macrophage-BMSC interactions under experimental conditions (e.g., DS-exo@A2M treatment ± IL-4 knockdown).

Detailed protocols for reagent preparation, imaging parameters, and statistical analyses are provided in the Supplementary Material.

### RT-qPCR

Total RNA was extracted using Trizol reagent (Thermo Fisher Scientific), reverse transcribed into cDNA with a specific kit (Beyotime), and quantified via RT-qPCR (Vazyme Biotech) under standardized thermal cycling conditions (95 °C denaturation, 60 °C annealing, 72 °C extension). Primer sequences (Table S2) were designed by Shanghai Sangon Biotech, and gene expression was normalized to GAPDH using the 2−ΔΔCt method. Detailed protocols, including reagent specifications and thermal cycling parameters, are provided in the Supplementary Material.

### Western blot

Protein lysates were prepared using RIPA buffer (Beyotime) with PMSF, and protein concentrations were quantified via BCA assay (Beyotime). Target proteins were separated by SDS-PAGE (8–12% gels), transferred to PVDF membranes (Bio-Rad), and detected using primary antibodies (Table S3) and HRP-conjugated secondary antibodies (Abcam/Cell Signaling). GAPDH served as a loading control, with relative protein expression analyzed via ImageJ software. Detailed antibody information and protocols are provided in Table S2 and the Supplementary Material.

### Establishment of ONFH rat model

A steroid-induced ONFH rat model was established via weekly intramuscular injections of methylprednisolone (20 mg/kg/day for 3 weeks). All animal experiments were approved by the Animal Ethics Committee of Fujian Medical University (IACUC FJMU 2024-0299). All animal experiments complied with the China Government animal experiment regulations and ARRIVE guidelines. Rats received tail vein injections of DS-exo@A2M (100 μg/200 μL) or saline post-treatment. To evaluate in vivo uptake of DS-exo@A2M by macrophages, rats were intravenously injected with Cy5.5-labeled DS-exo@A2M via the tail vein. At designated time points, bone marrow-derived macrophages were isolated and subjected to flow cytometry to detect Cy5.5 fluorescence intensity. Serum biomarkers (CK, LDH, ALT, AST, CREA, urea) and femoral tissues were analyzed post-euthanasia. Detailed group allocations, dosing regimens, and tissue collection protocols are provided in the Supplementary Material.

### Statistical analysis

In our study, the bioinformatics results were subjected to statistical analysis using R 4.2.1, while the remaining data were analyzed using SPSS 26.0 (IBM, USA). Continuous data are presented as mean ± standard deviation. Normality and homogeneity of variance were assessed initially. In cases where both conditions were met, an independent samples *t*-test or paired samples t-test was conducted for between-group comparisons, and for multiple group comparisons, ANOVA or repeated measures ANOVA were employed. A significance level of *p* < 0.05 was considered indicative of statistical significance.

## Supplementary information

supplementary material

Full and uncropped western blots

## Data Availability

All data can be provided as needed.
