# Supplementary material for: Metabolic glycoengineered exosome-A2M nanoplatform reprograms macrophage polarization and orchestrates bone regeneration in ONFH
Source: Cell Death Discov. 2025 Nov 7;11:510. doi: 10.1038/s41420-025-02690-8 (PMC12594842; doi:10.1038/s41420-025-02690-8)
Supplement: Supplementary file 1 — supplementary material [file 41420_2025_2690_MOESM1_ESM.docx]

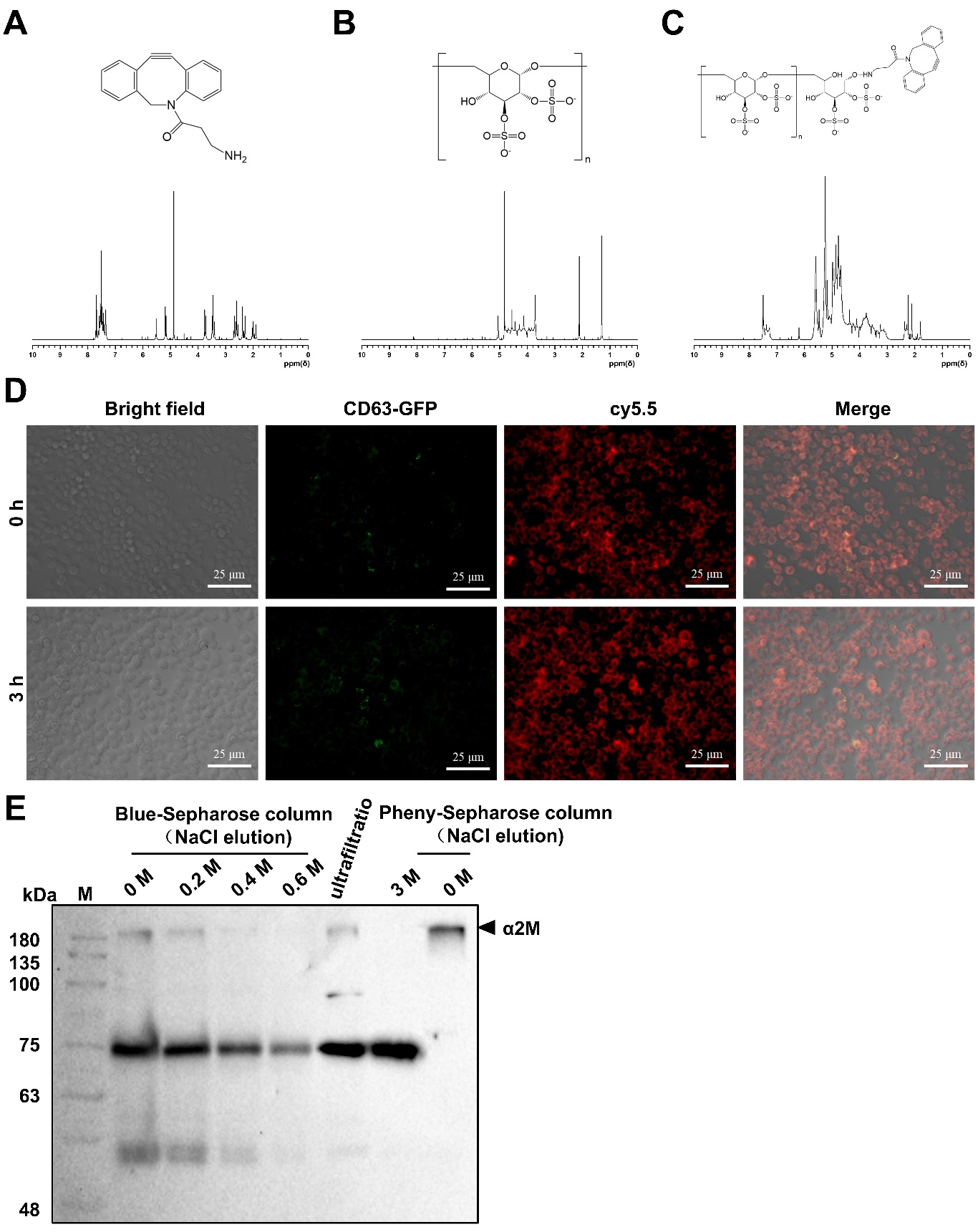


**Fig. S1. Characterization of Engineered Exosomes Loaded with A2M.**

Note: (A-C) Chemical structures and 1H NMR spectra of DBCO-amine, DS, and DBCO-DS; (D) Confocal microscopy image showing CD63-GFP (green) and cy5.5-labeled DS (red) in macrophages (scale bar = 25 μm,); (E) Western Blot detection of purified A2M; all cellular experiments were repeated three times.


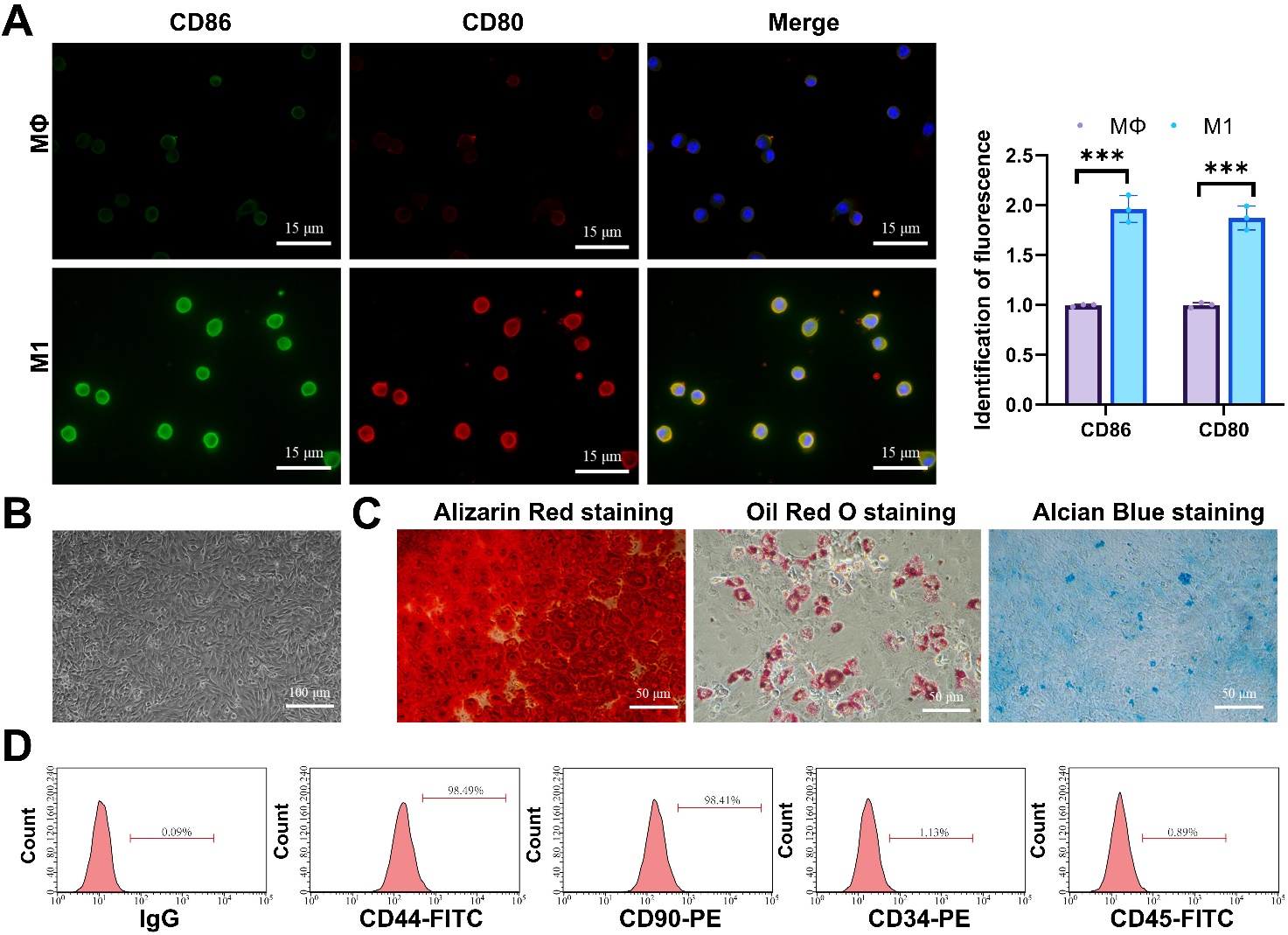


**Fig. S2. Identification of Macrophages and BMSCs.**

Note: (A) Validation of Macrophage (MΦ) differentiation (scale bar = 15 μm); (B) Observation of BMSCs morphology under inverted microscope (scale bar = 100 μm); (C) Evaluation of osteogenic, adipogenic, and chondrogenic differentiation abilities of BMSCs through Safranin-O, Oil Red O, and Alcian Blue staining (scale bars = 25 μm); (D) Flow cytometry analysis of BMSCs marker expression. ****p* < 0.001; all cell experiments were performed in triplicate.


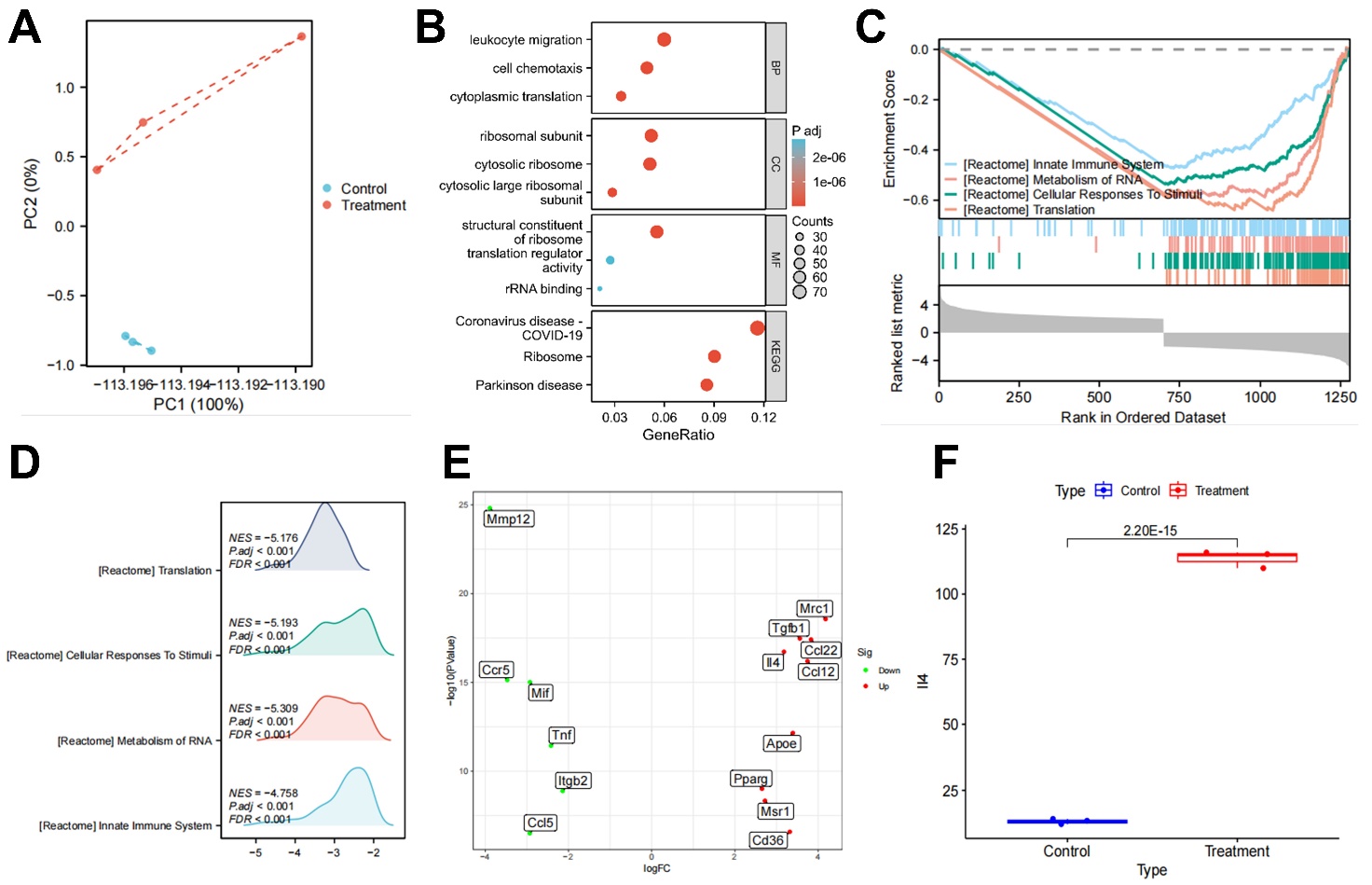


**Fig. S3. RNA-seq Data Analysis.**

Note: (A) PCA of RNA-seq data; (B) Bubble plots showing enrichment analysis of DEGs in GO and KEGG; (C) Results of GSEA for DEGs; (D) Landscape plot of GSEA analysis for DEGs; (E) Volcano plot displaying gene expression of 15 M2-DEGs, with significant upregulated genes represented by red dots and downregulated genes by green dots; (F) Expression pattern of IL-4 in RNA-seq data; Sample size: n = 3 for each group.


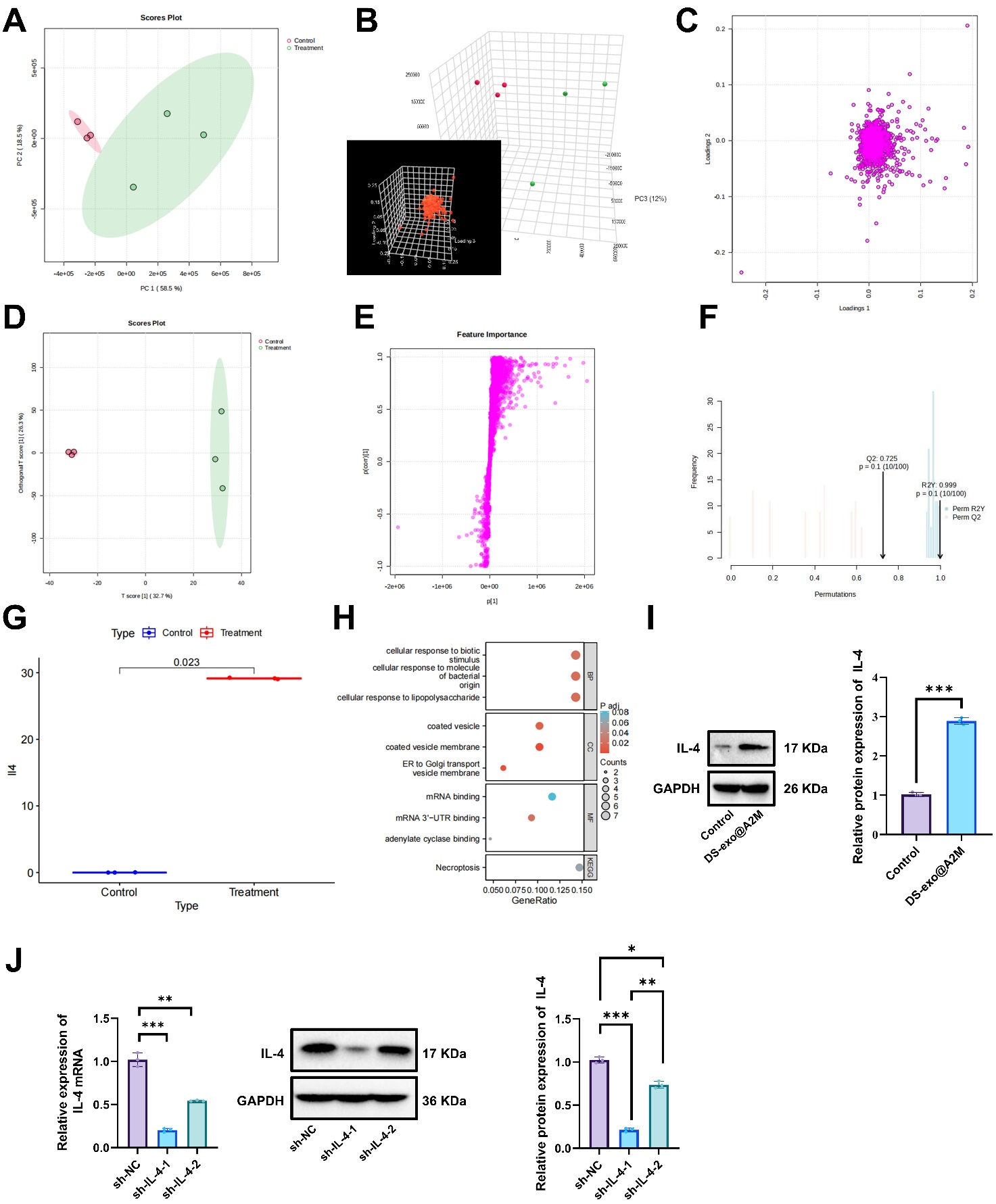


**Fig. S4. Protein Sequencing Data Analysis.**

Note: (A) PCA plot of proteomic data; (B) 3D-PCA plot of proteomic data; (C) Loading plot of proteomic data; (D) OPLS-DA plot of proteomic data; (E) S-plot of proteomic data; (F) Permutation plot of proteomic data; (G) Expression of IL-4 in proteomic data; (H) Bubble plots for GO and KEGG enrichment analysis of differentially expressed proteins; (I) RT-qPCR and Western Blot analysis of IL-4 expression levels in each group; (J) RT-qPCR and Western Blot validation of IL-4 silencing efficiency in macrophages without exosome loading. Each group of protein sequencing data consists of n = 3 samples. Significance levels: ***p* < 0.01, ****p* < 0.001. All cell experiments were performed in triplicate.


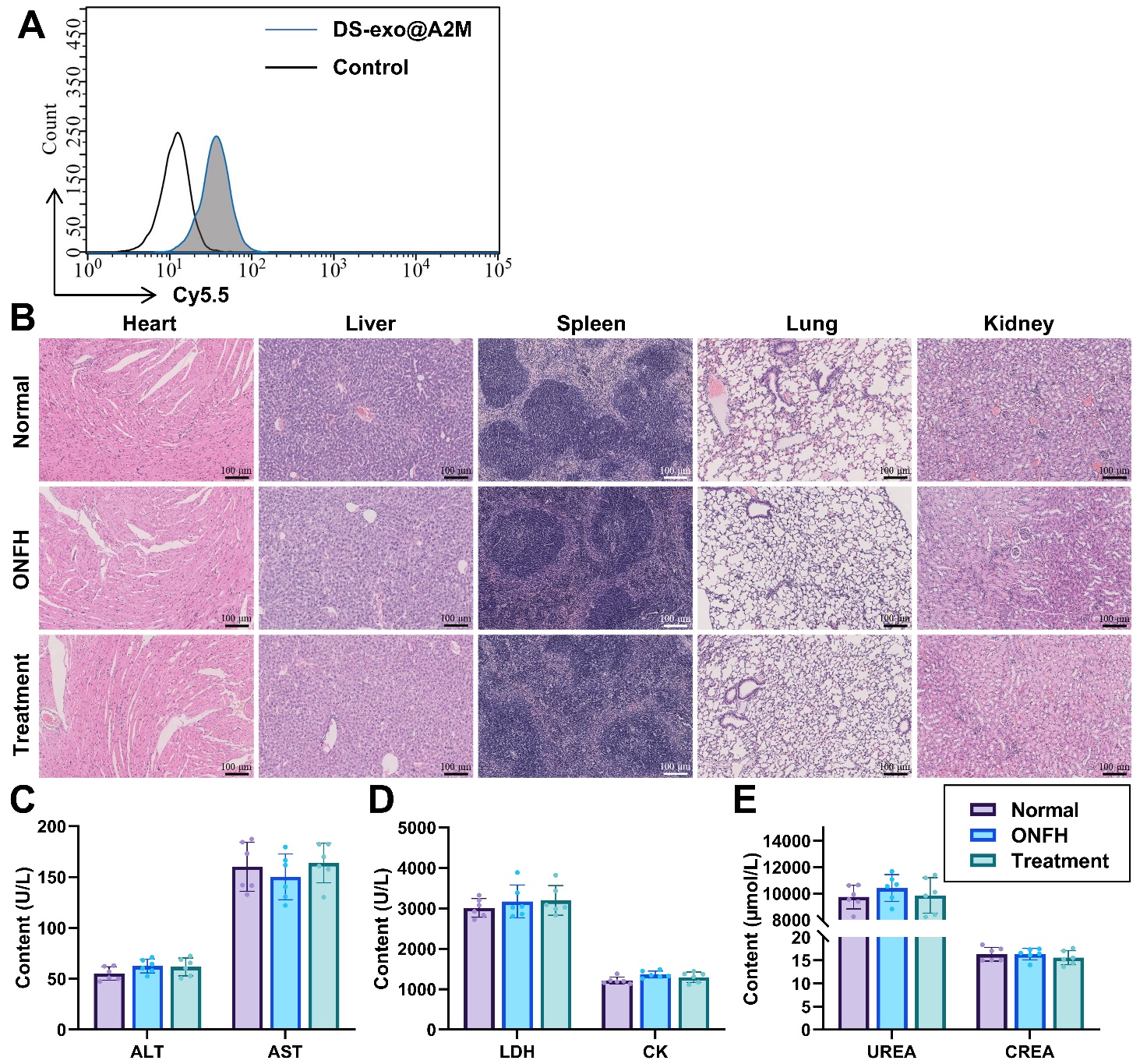


**Fig. S5. *In Vivo* Biosafety of DS-exo@A2M.**

Note: (A) Flow cytometric analysis of *in vivo* uptake of DS-exo@A2M by macrophages in rats; (B) Histopathology Results of Rat Heart, Liver, Spleen, Lung, and Kidney (scar = 100 μm); (C) Assessment of Liver Function Parameters (ALT, AST) in Different Rat Groups; (D) Evaluation of Cardiac Function Parameters (CK, LDH) in Different Rat Groups; (E) Analysis of Renal Function Parameters (CREA, urea) in Different Rat Groups; n = 6 rats per group.

**Table S1. Sequence of RT-qPCR**

| **Name** | **Sequence (5’-3’)** |
| --- | --- |
| CD163 (Rat) | F: TCCGGTTGAAGTTTTGTGACC |
|  | R: GTGGTCCCGATGACCGTATT |
| IL-10 (Rat) | F: CCTCTGGATACAGCTGCGAC |
|  | R: GTAGATGCCGGGTGGTTCAA |
| CD80 (Rat) | F: CTGTCAAATTCCGACGCTGC |
|  | R: GGAAGGCAAATGGCTACCTT |
| CD86(Rat) | F: GAGCTGGTAGTATTTTGGCAGG |
|  | R: GGCCCAGGTACTTGGCATT |
| IL-1β(Rat) | F: GACTTCACCATGGAACCCGT |
|  | R: GGAGACTGCCCATTCTCGAC |
| IL-4 (Rat) | F: CGTGATGTACCTCCGTGCTT |
|  | R: GTGAGTTCAGACCGCTGACA |
| Runx2(Rat) | F: CACAAGTGCGGTGCAAACTT |
|  | R: AAGAGGCTGTTTGACGCCAT |
| Osterix(Rat) | F: CAGTAATCTTCGTGCCAGACCT |
|  | R: TAGTGAGCTTCTTCCTGGGGA |
| Alpl(Rat) | F: CTCCTTAGGGCCACCGCT |
|  | R: AGCCGTTAATTGACGTTCCG |
| Opn(Rat) | F: GATGACGACGACGATGACGA |
|  | R: GCTGGCAGTGAAGGACTCAT |
| Ocn(Rat) | F: GAATAGACTCCGGCGCTACC |
|  | R: TCCTGGAAGCCAATGTGGTC |
| GAPDH (Rat) | F: CTCAGTTGCTGAGGAGTCCC |
|  | R: ATTCGAGAGAAGGGAGGGCT |

Abbreviations: F, forward; R, reverse

**Table S2. Primary antibody information**

| **Name** | **Cat.NO** | **Dilution ratio** | **Manufactor** | **Country** |
| --- | --- | --- | --- | --- |
| HSP70 (anti-mouse) | ab2787 | 1: 1000 | Abcam | UK |
| CD9(anti-rabbit) | ab307085 | 1: 1000 | Abcam | UK |
| CD63(anti-mouse) | 67605-1-Ig | 1: 5000 | Proteintech | China |
| TGS101(anti-rabbit) | ab125011 | 1: 1000 | Abcam | UK |
| Calnexin(anti-rabbit) | ab22595 | 1 µg/mL | Abcam | UK |
| RUNX2(anti-mouse) | ab76956 | 1: 1000 | Abcam | UK |
| ALPL(anti-mouse) | ab65834 | 1: 1000 | Abcam | UK |
| A2M (anti-rabbit) | ab316101 | 1: 1000 | Abcam | UK |
| IL-4(anti-mouse) | 66142-1-Ig | 1: 1000 | Proteintech | China |
| GAPDH (anti-rabbit) | ab181602 | 1: 10000 | Abcam | UK |

**Table S3. PPI network data obtained from the second screening**

| Name | Betweenness | Closeness | Degree | Eigenvector | LAC | Network |
| --- | --- | --- | --- | --- | --- | --- |
| Ccl5 | 0 | 1 | 5 | 0.408248276 | 4 | 5 |
| Ccr5 | 0 | 1 | 5 | 0.408248305 | 4 | 5 |
| Cd36 | 0 | 1 | 5 | 0.408248276 | 4 | 5 |
| Tgfb1 | 0 | 1 | 5 | 0.408248276 | 4 | 5 |
| Mrc1 | 0 | 1 | 5 | 0.408248276 | 4 | 5 |
| Il4 | 0 | 1 | 5 | 0.408248276 | 4 | 5 |
