# Supplementary material for: Metabolic glycoengineered exosome-A2M nanoplatform reprograms macrophage polarization and orchestrates bone regeneration in ONFH
Source: Cell Death Discov. 2025 Nov 7;11:510. doi: 10.1038/s41420-025-02690-8 (PMC12594842; doi:10.1038/s41420-025-02690-8)

# Full and uncropped western blots

## Full and uncropped western blot of Figure 1C-1.jpg


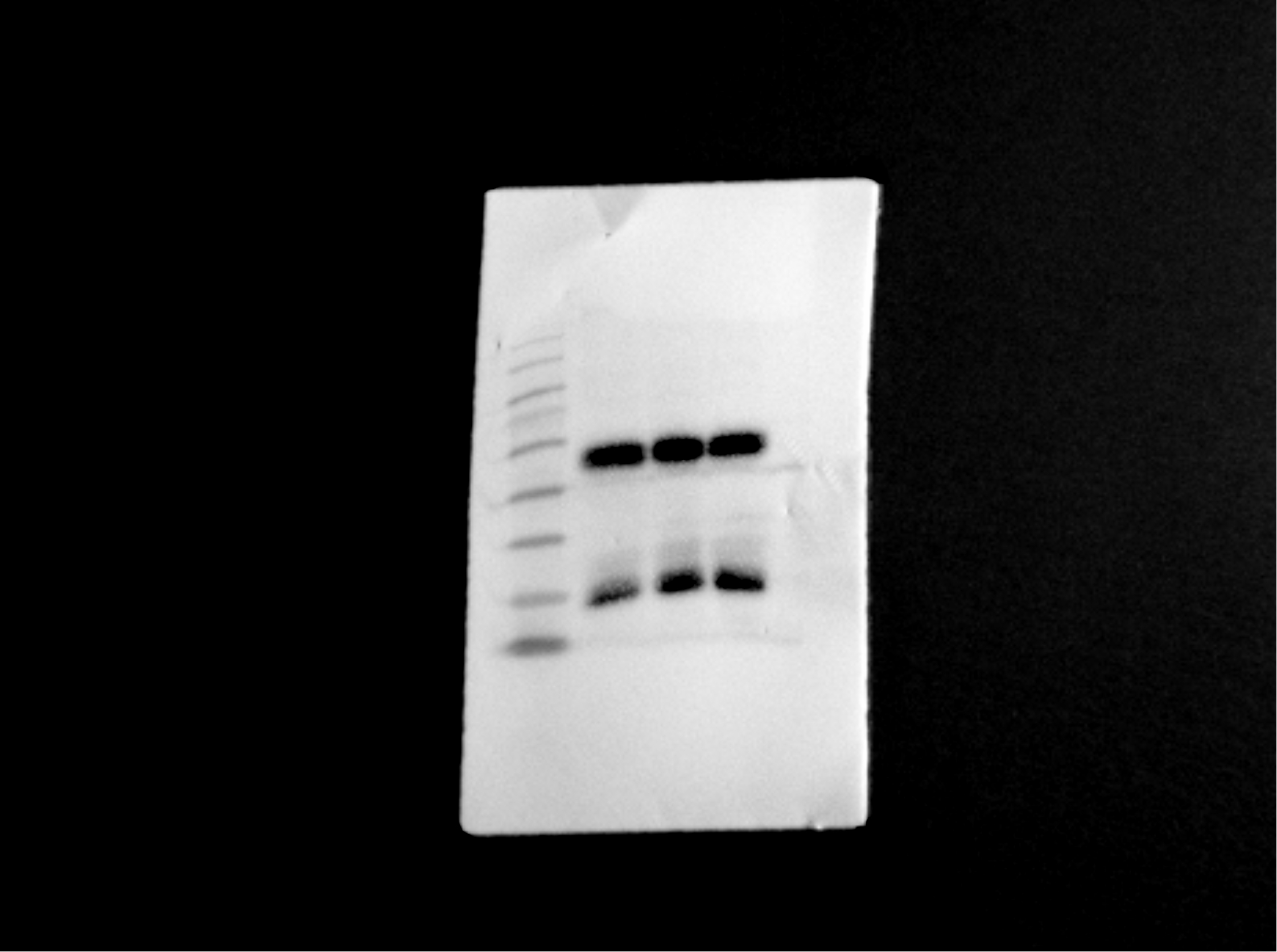


## Full and uncropped western blot of Figure 1C-2.jpg


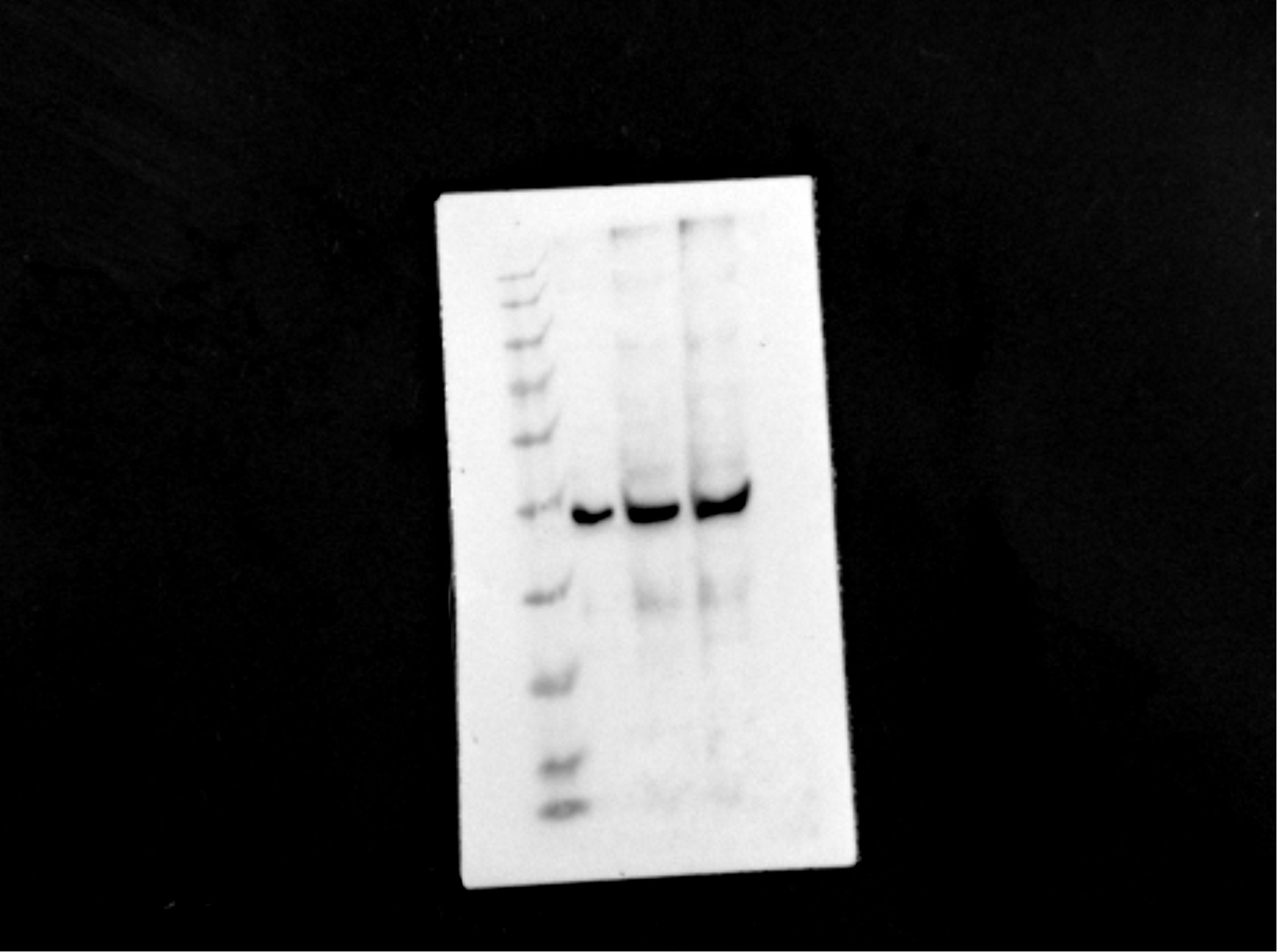


## Full and uncropped western blot of Figure 1C-3.jpg


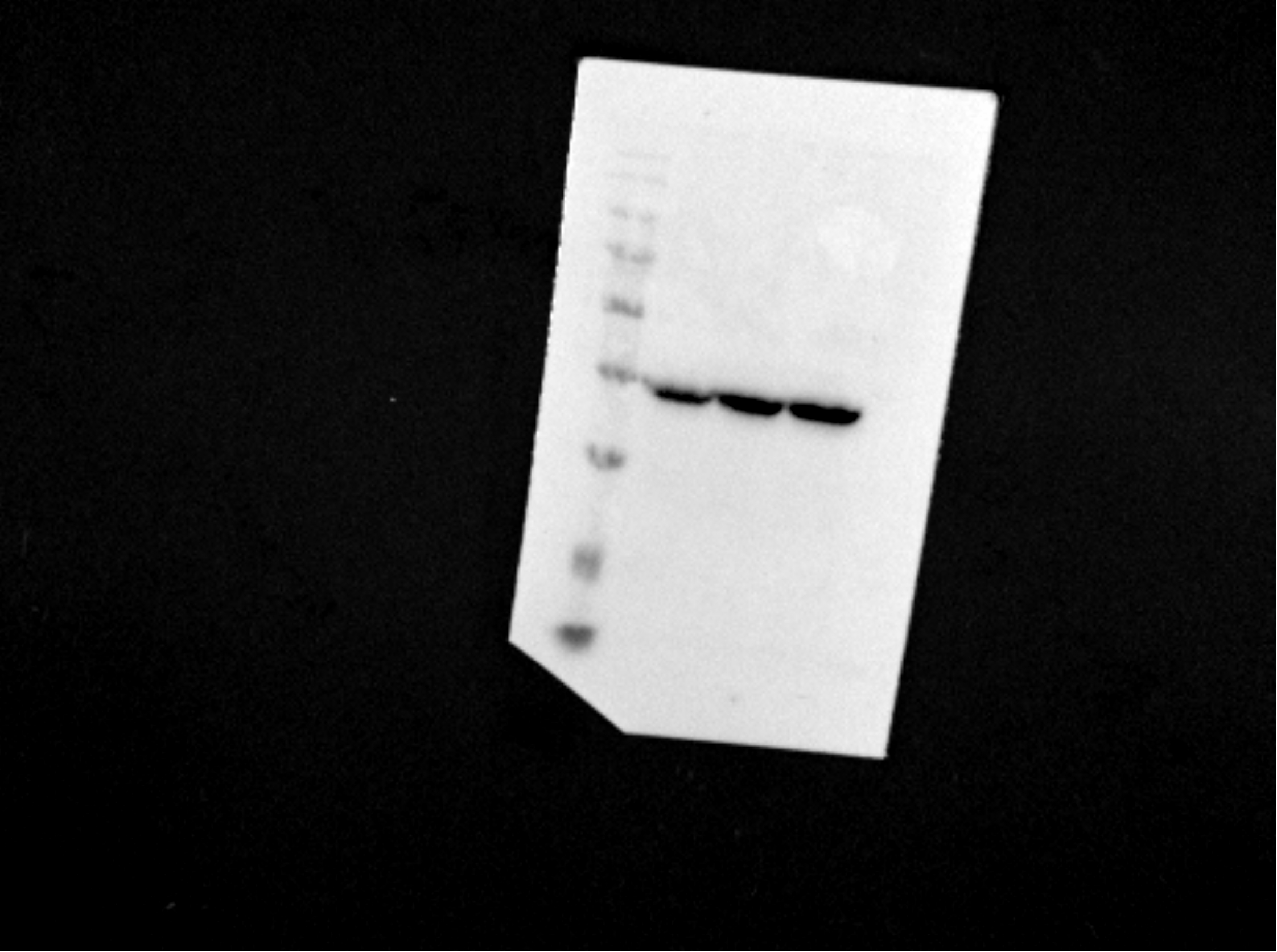


## Full and uncropped western blot of Figure 1C-4.jpg


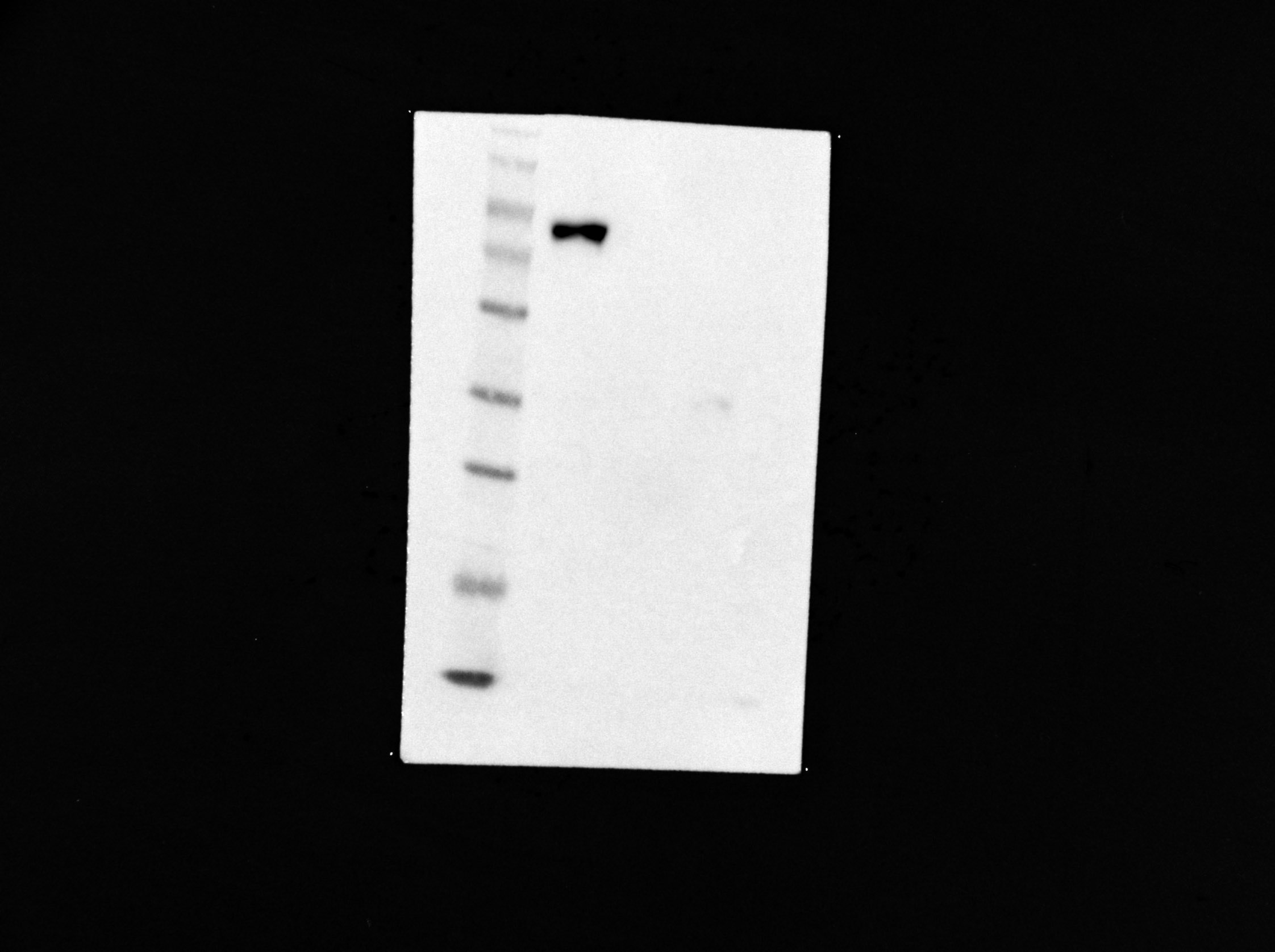


## Full and uncropped western blot of Figure 1J-1.jpg


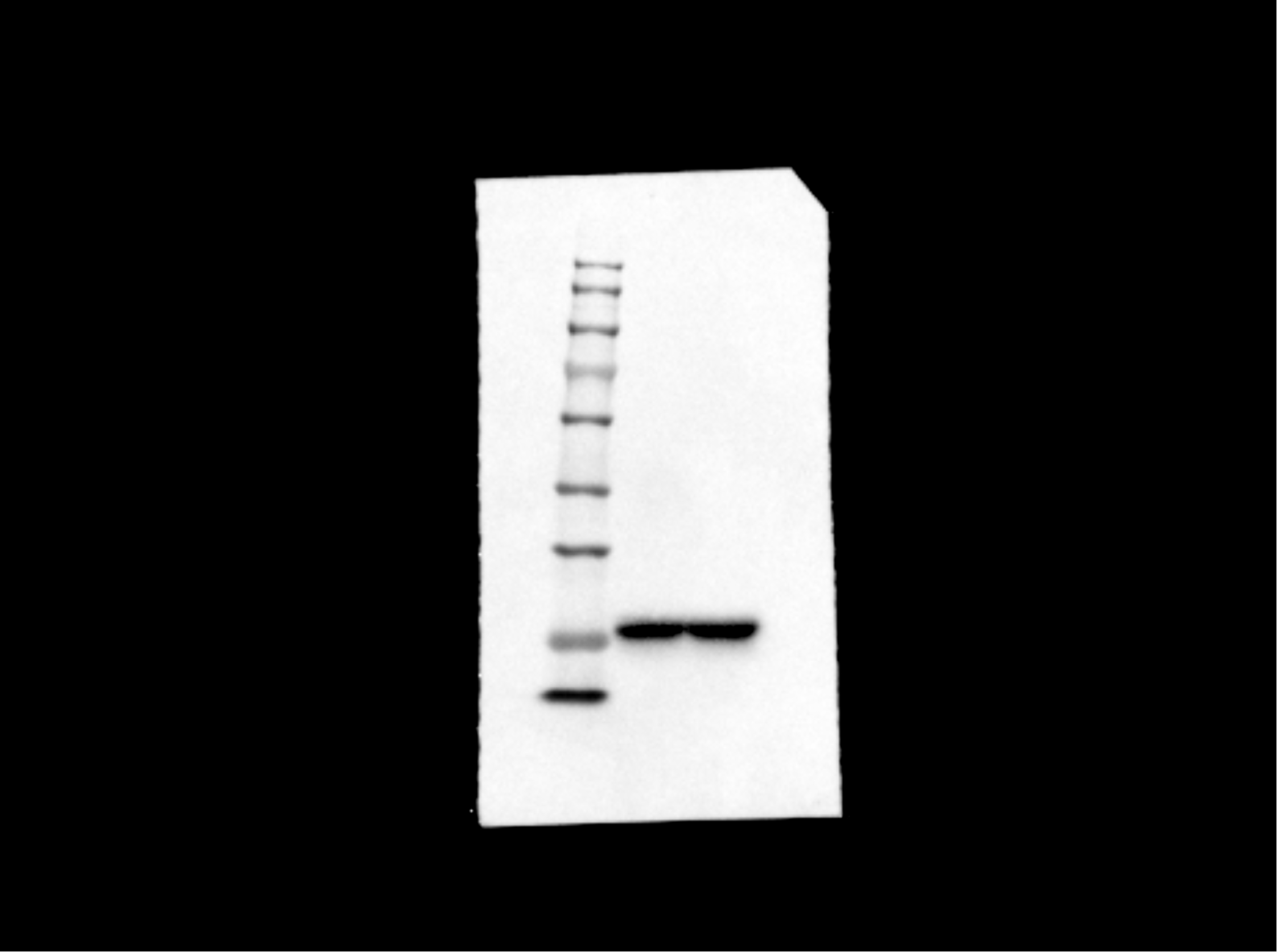


## Full and uncropped western blot of Figure 1J-2.jpg


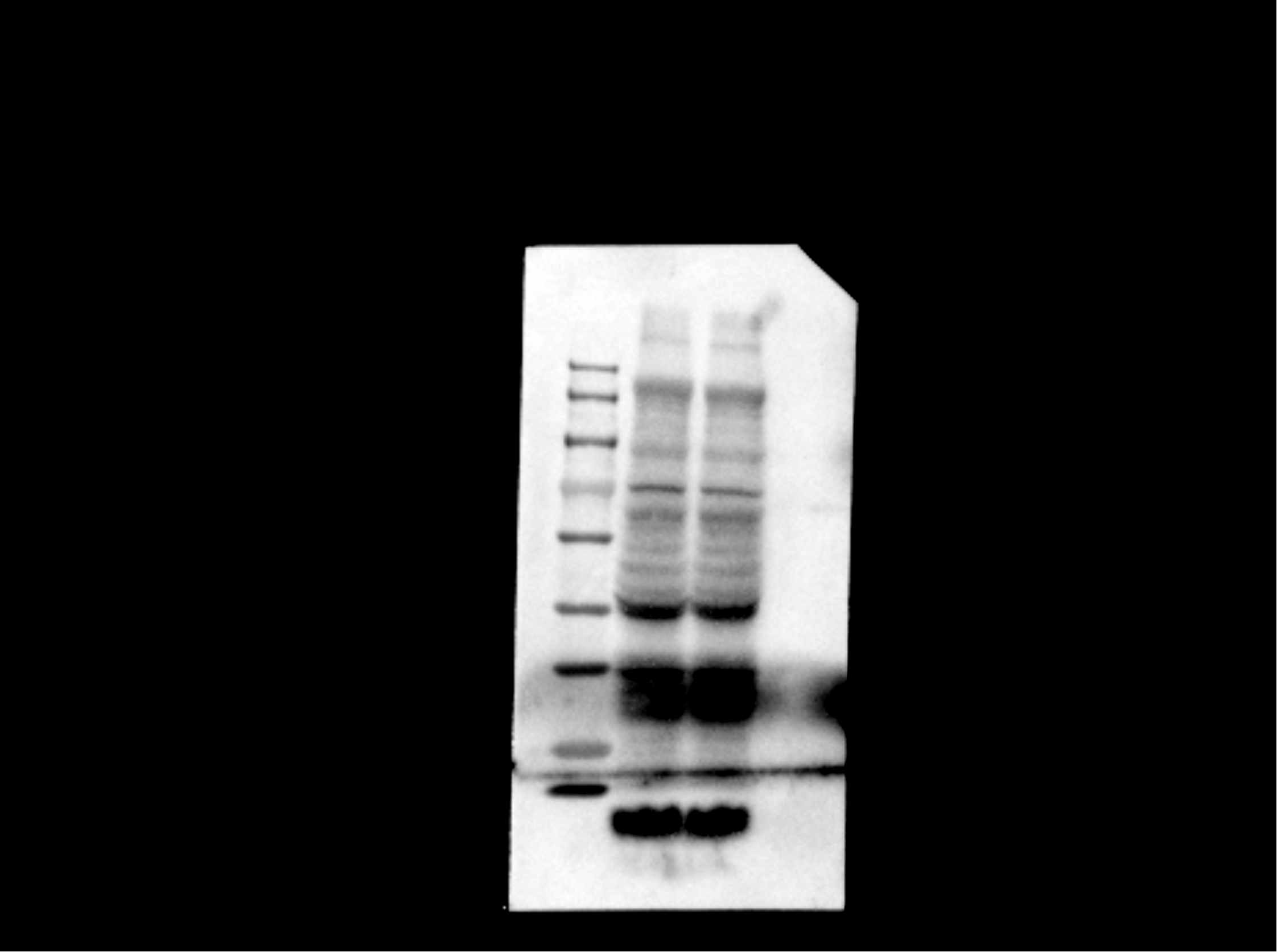


## Full and uncropped western blot of Figure 1J-3.jpg


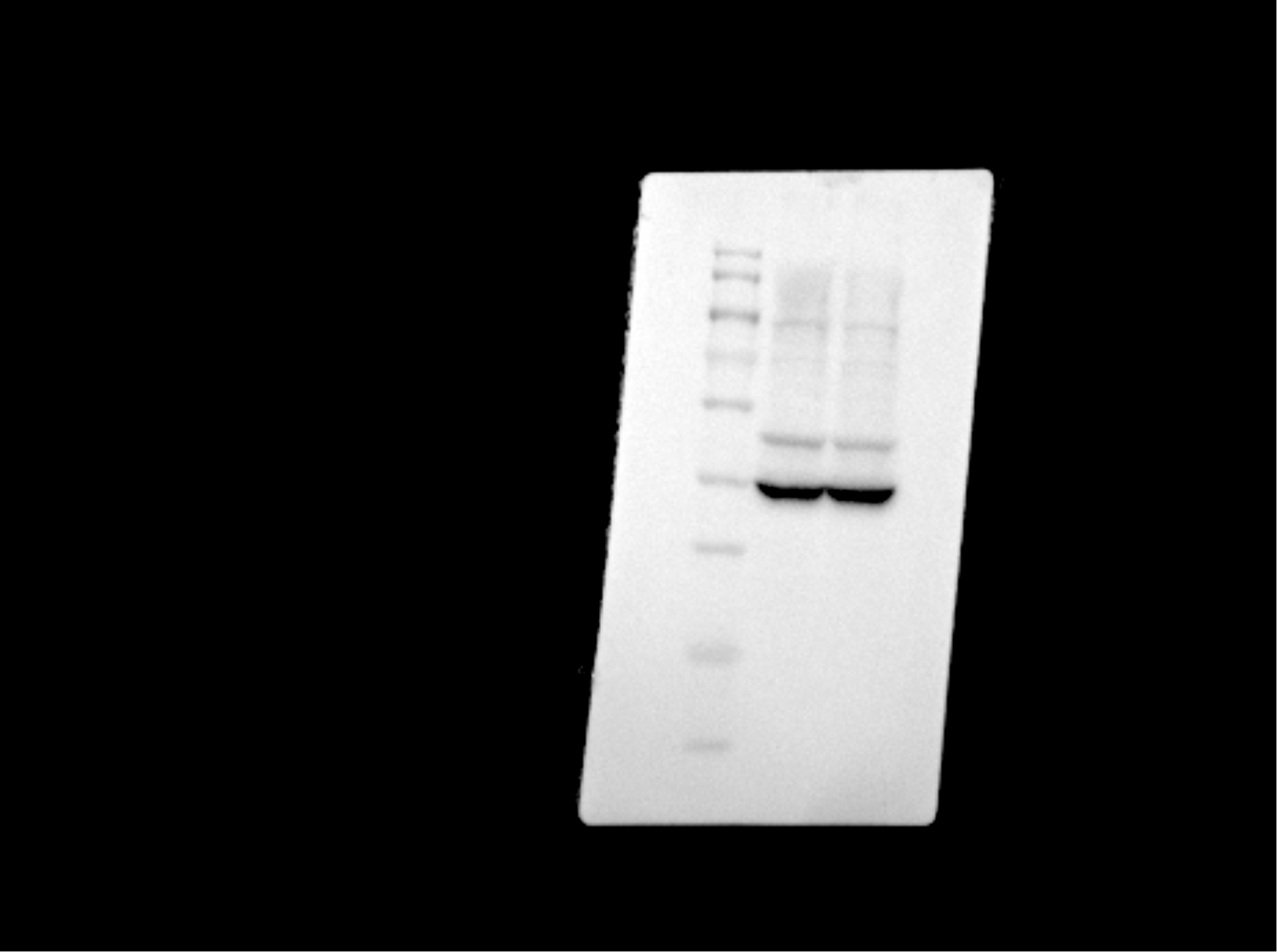


## Full and uncropped western blot of Figure 1J-4.jpg


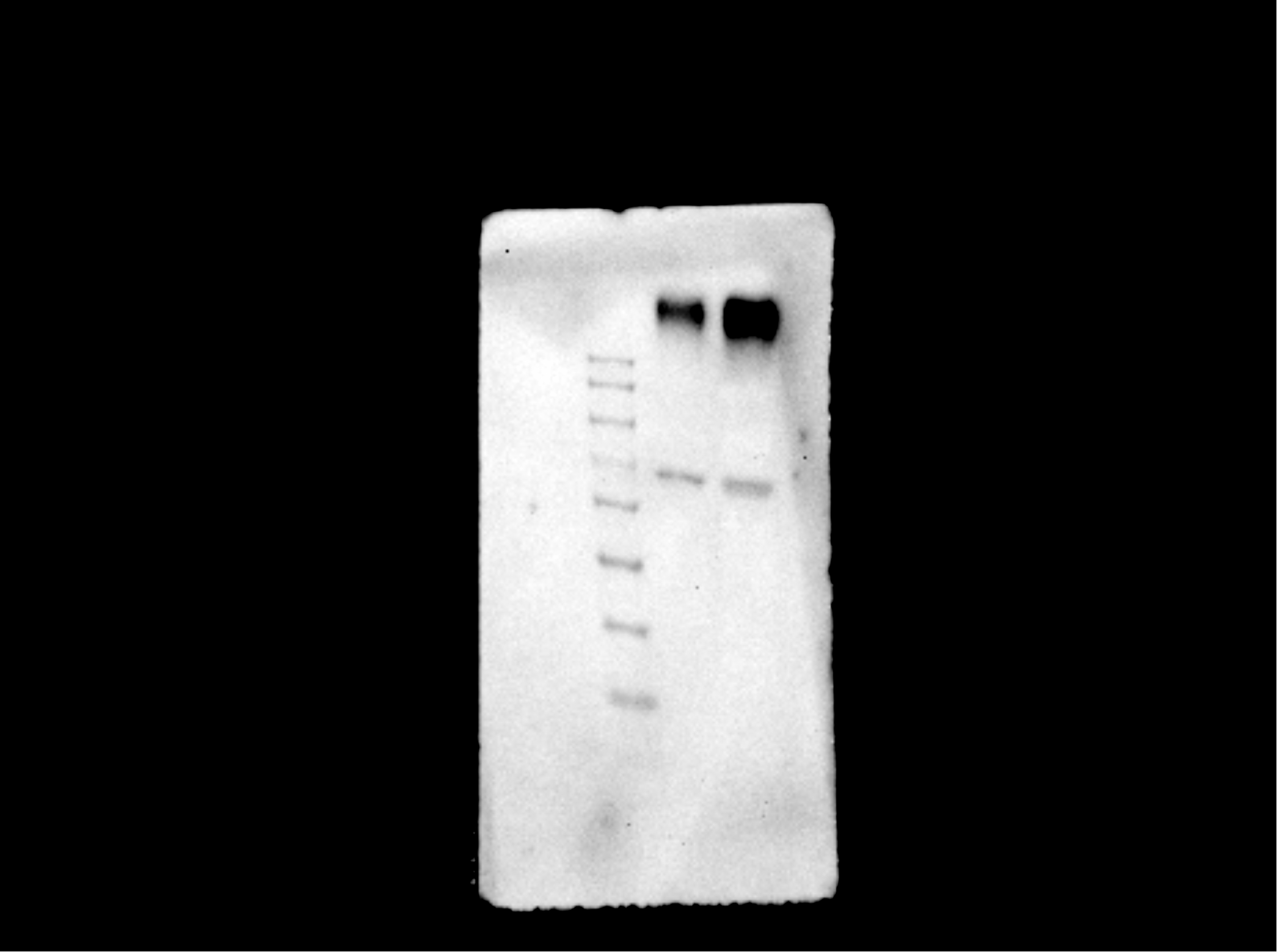


## Full and uncropped western blot of Figure 2I-1.jpg


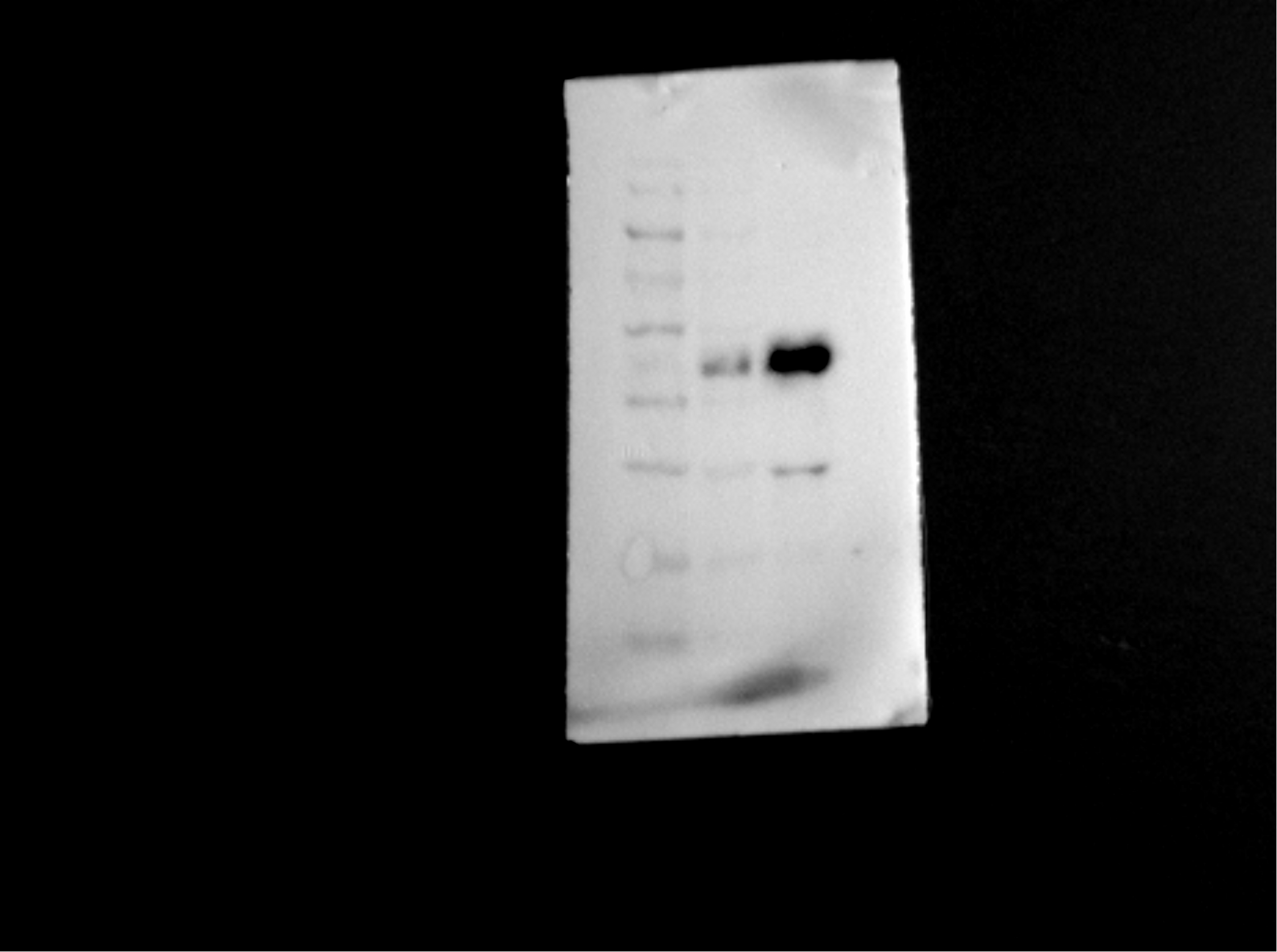


## Full and uncropped western blot of Figure 2I-2.jpg


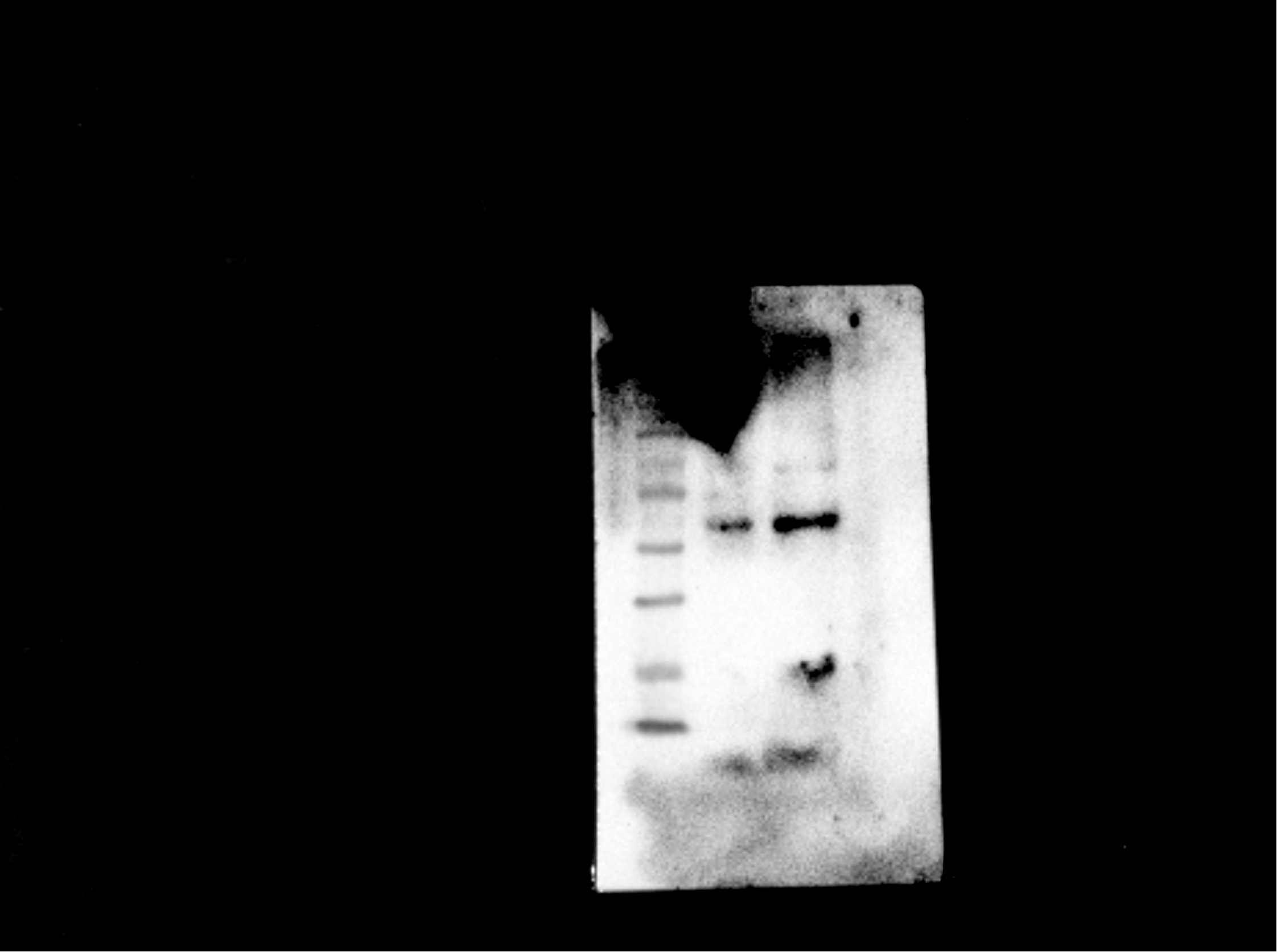


## Full and uncropped western blot of Figure 2I-3.jpg


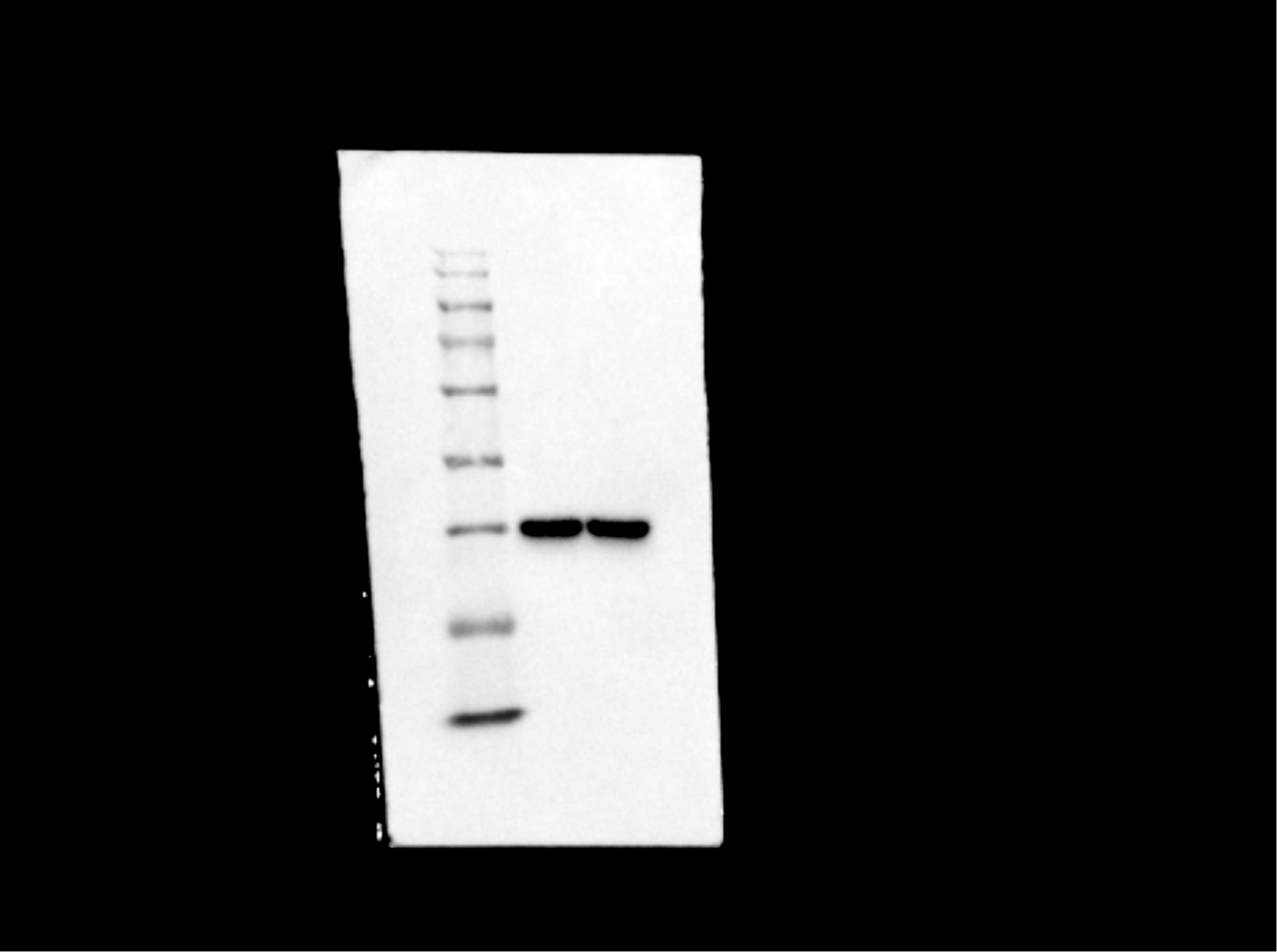


## Full and uncropped western blot of Figure 4H-1.jpg


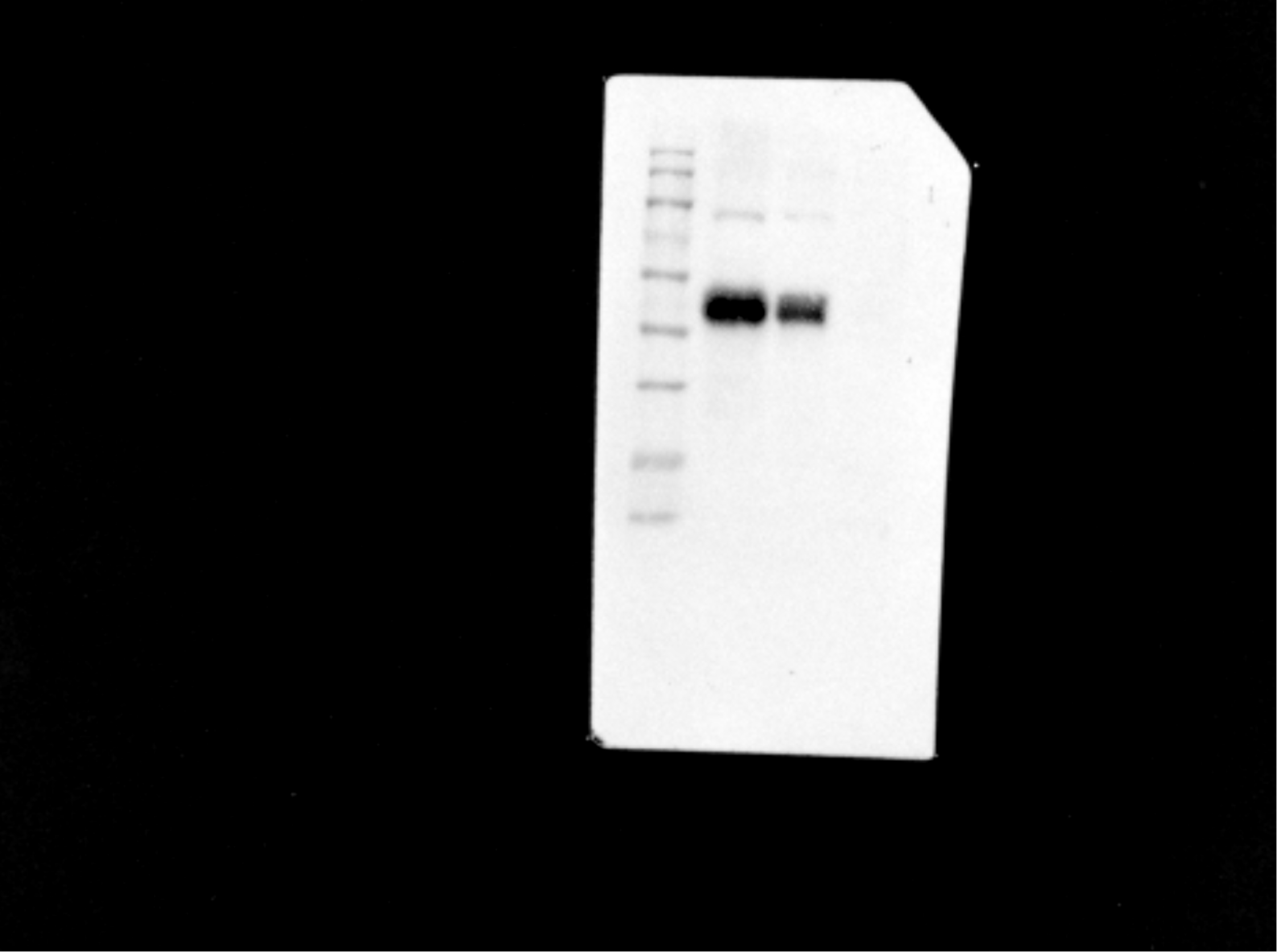


## Full and uncropped western blot of Figure 4H-2.jpg


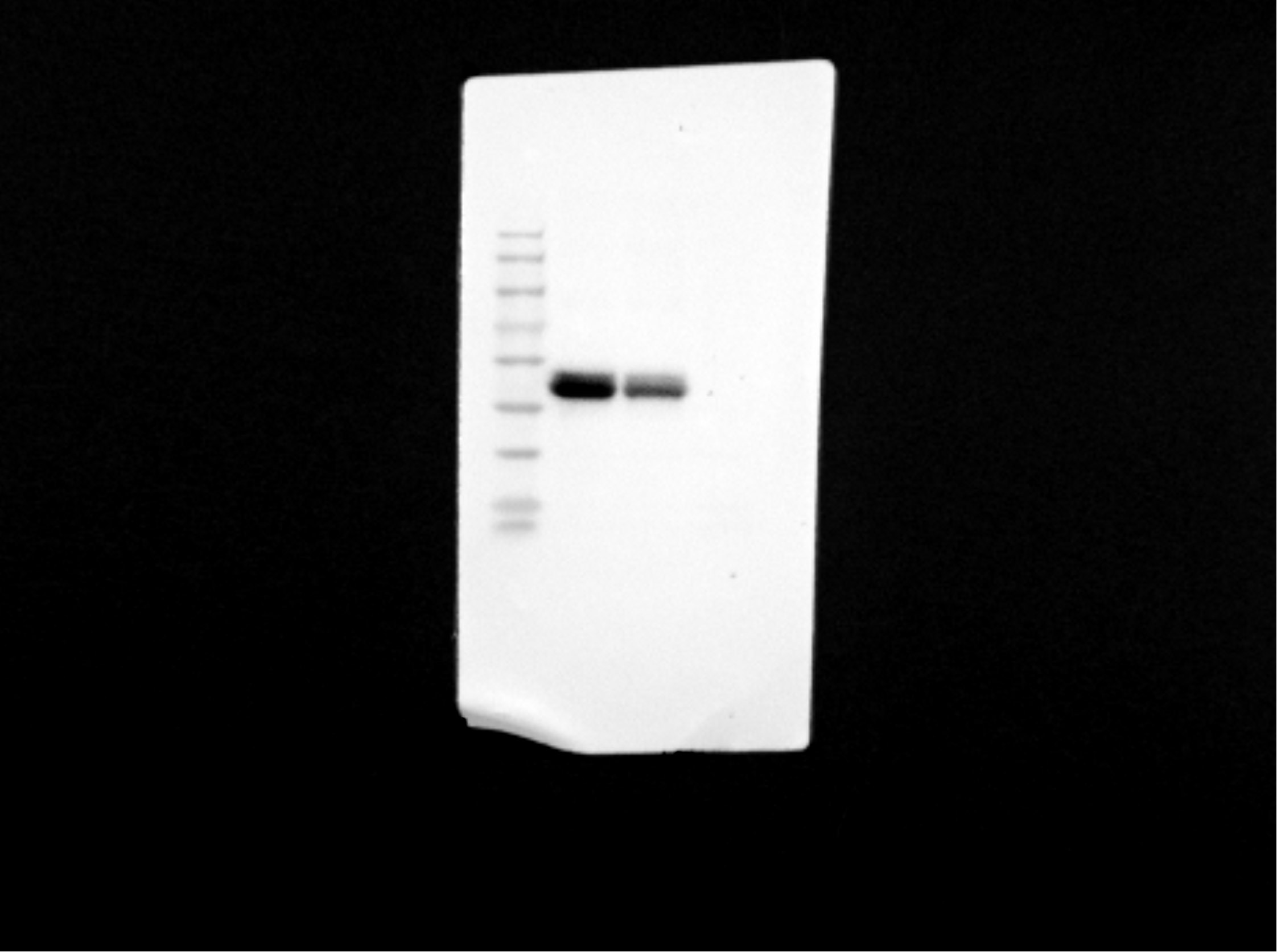


## Full and uncropped western blot of Figure 4H-3.jpg


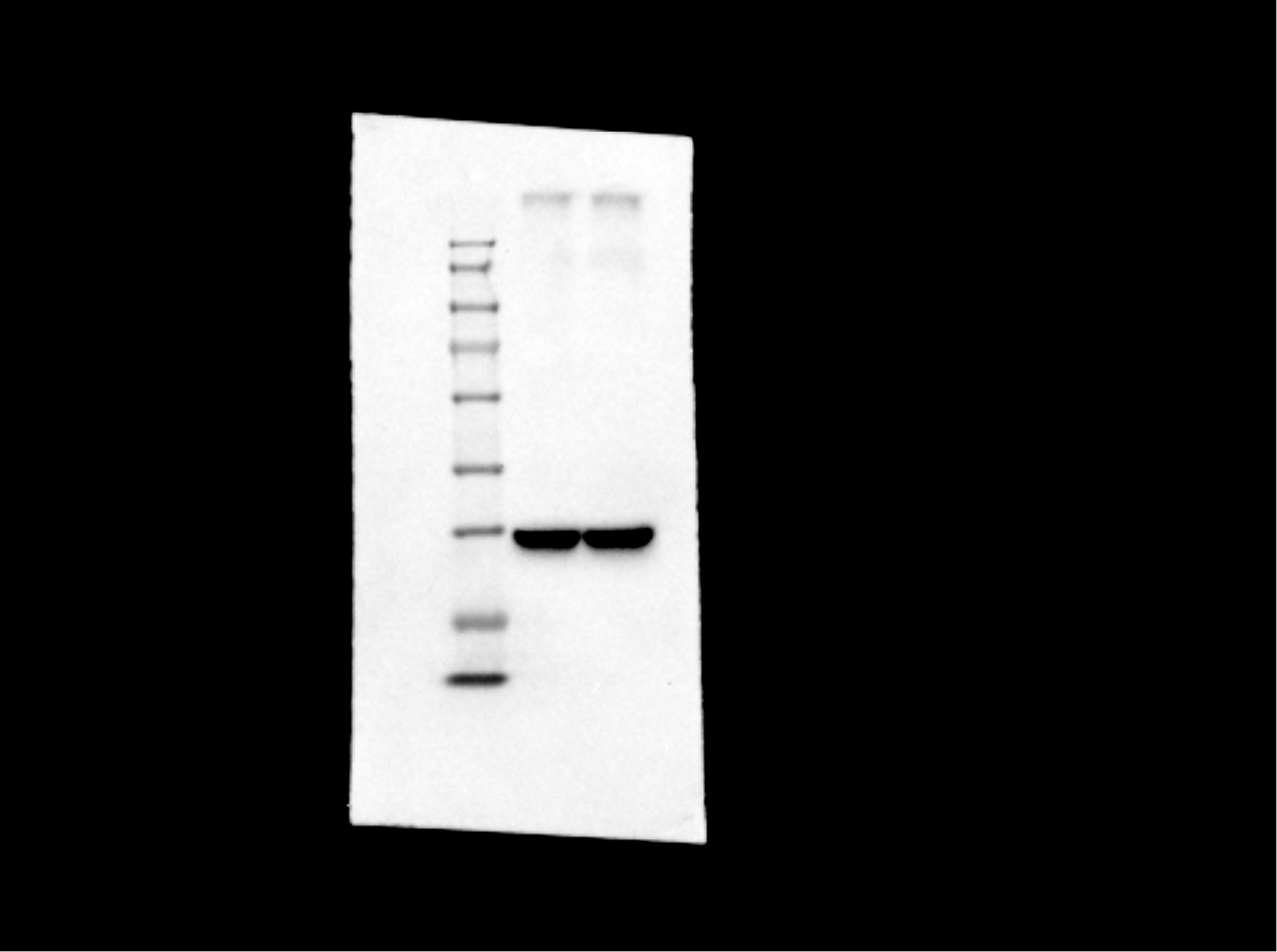


## Full and uncropped western blot of Figure 7D-1.jpg


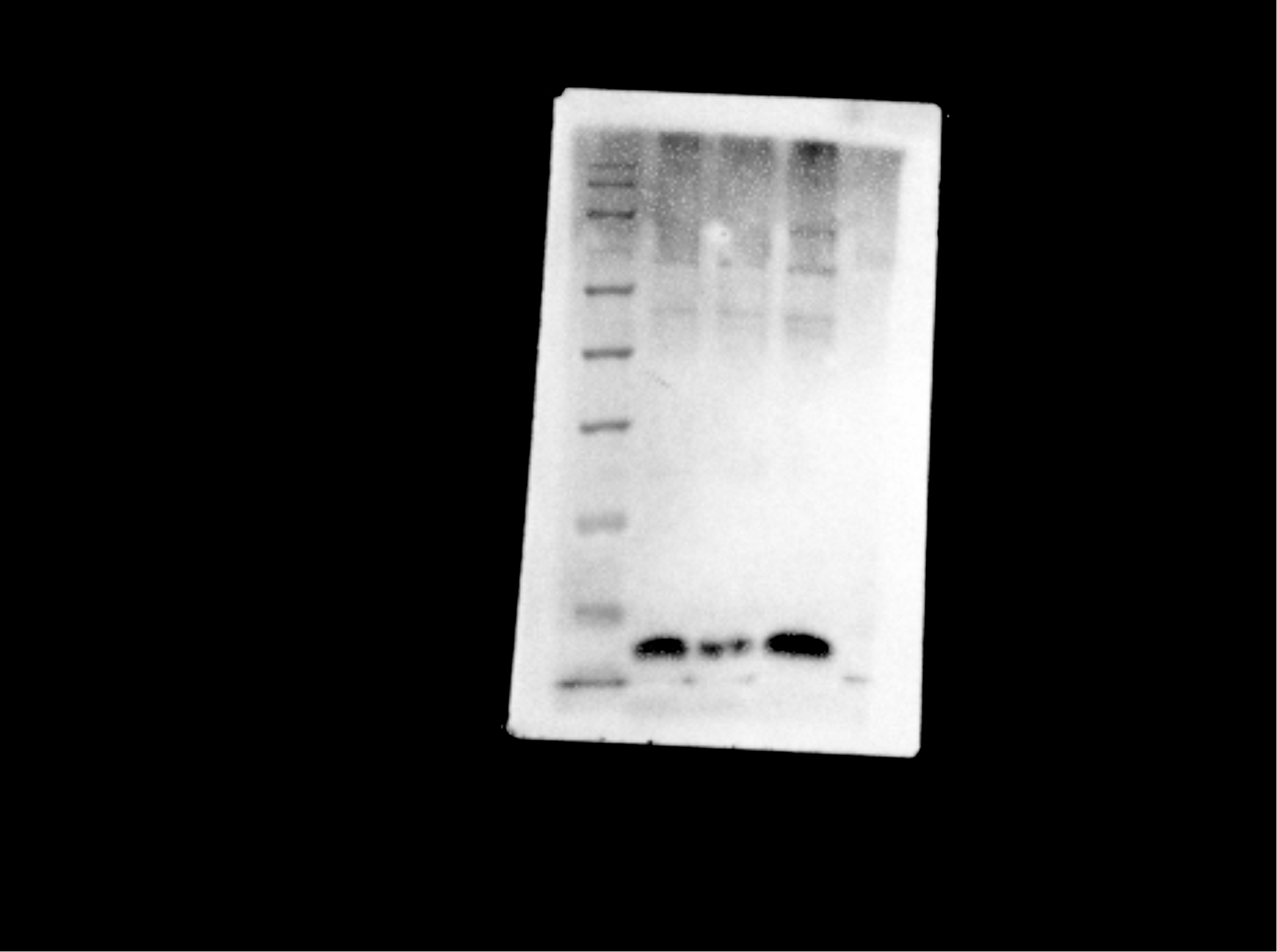


## Full and uncropped western blot of Figure 7D-2.jpg


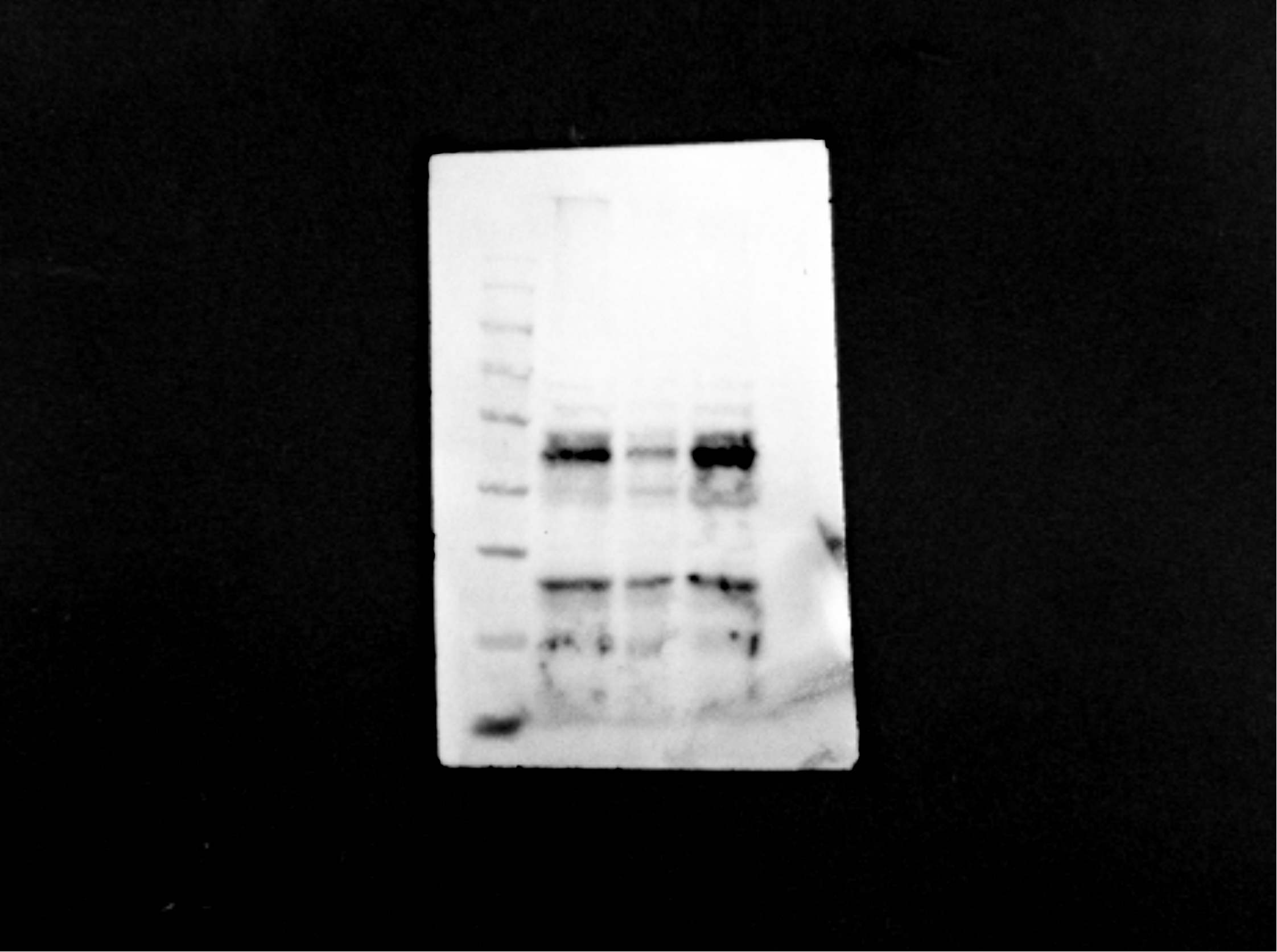


## Full and uncropped western blot of Figure 7D-3.jpg


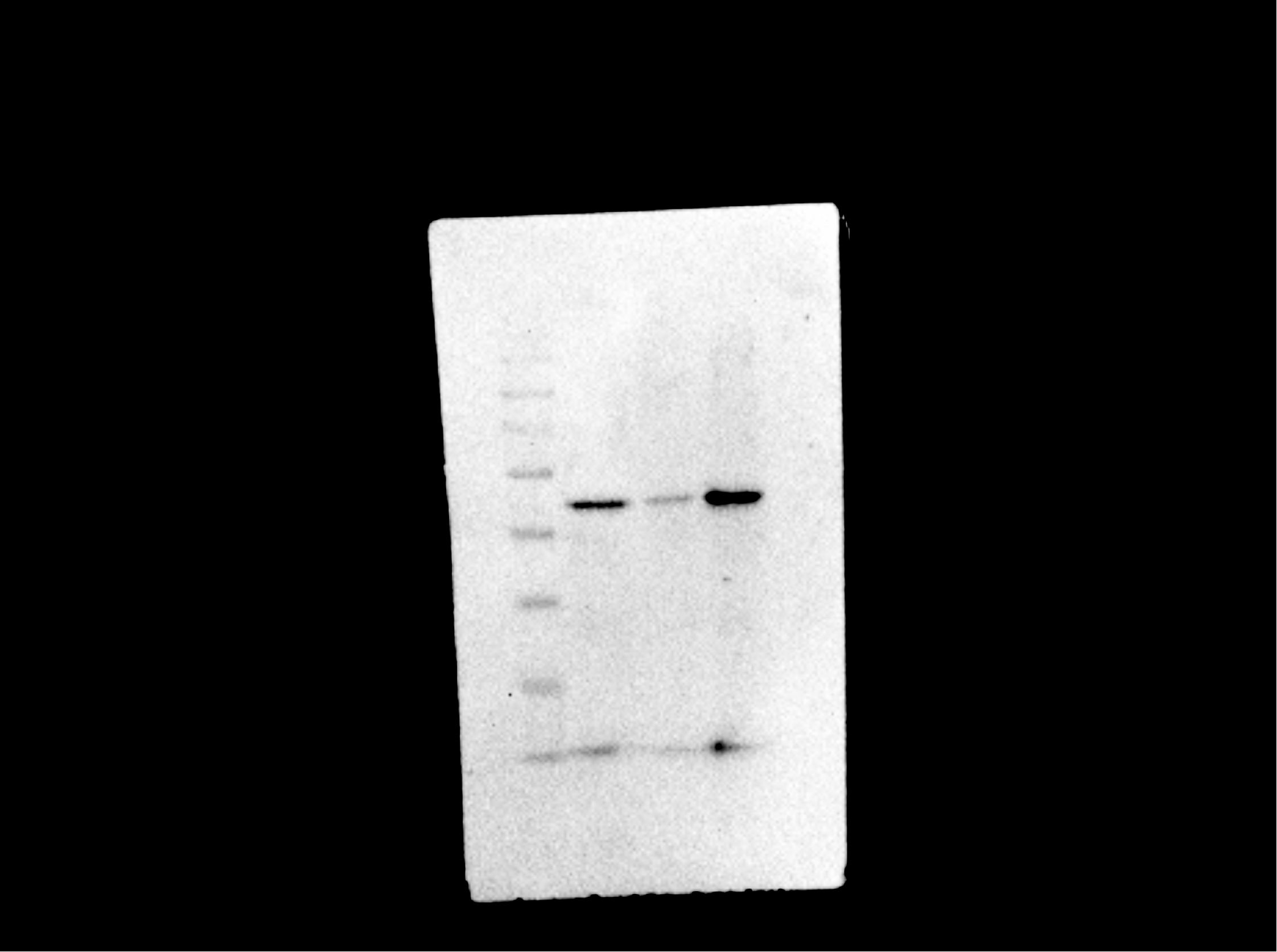


## Full and uncropped western blot of Figure 7D-4.jpg


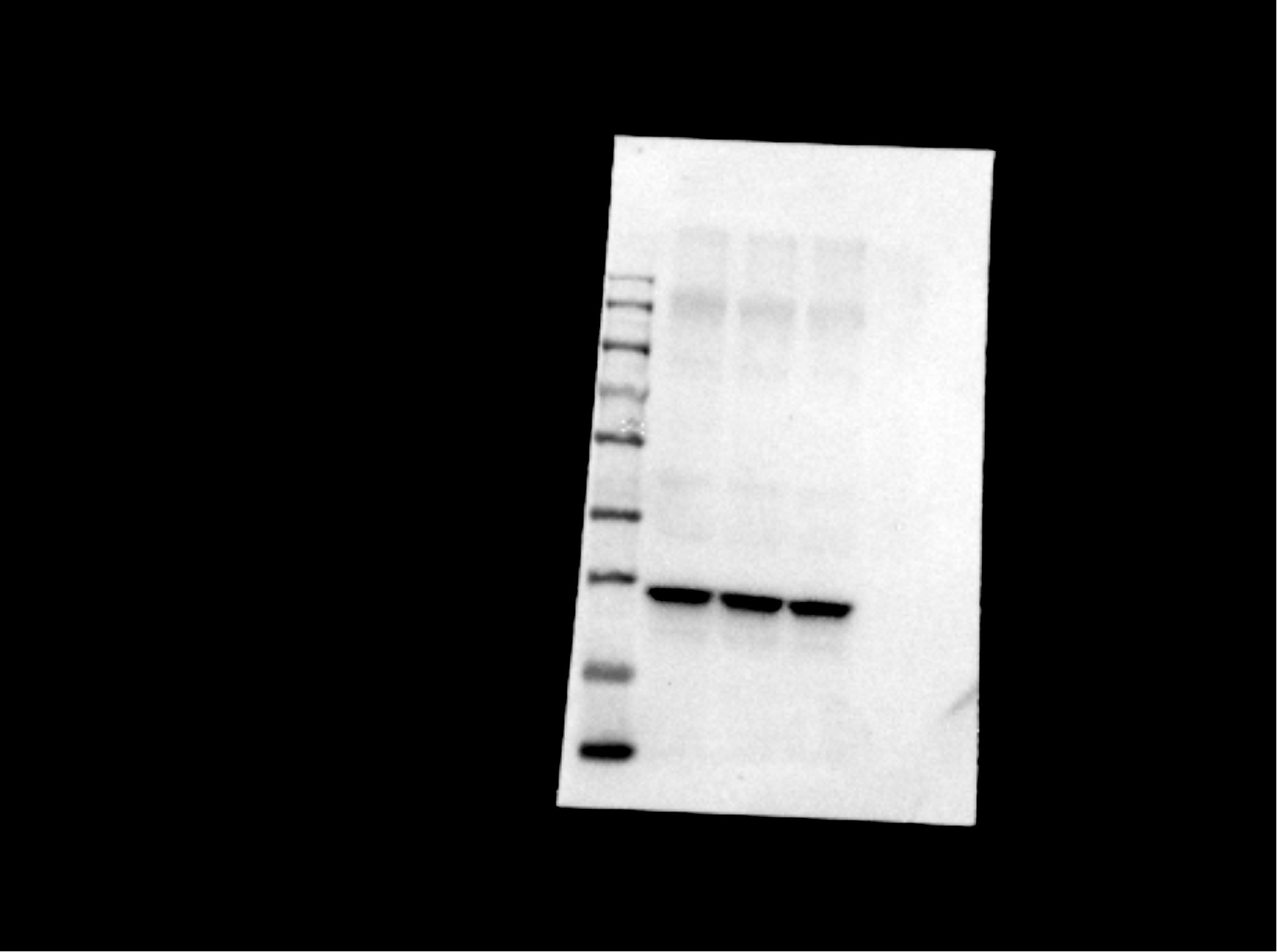


## Full and uncropped western blot of Figure S1E.jpg


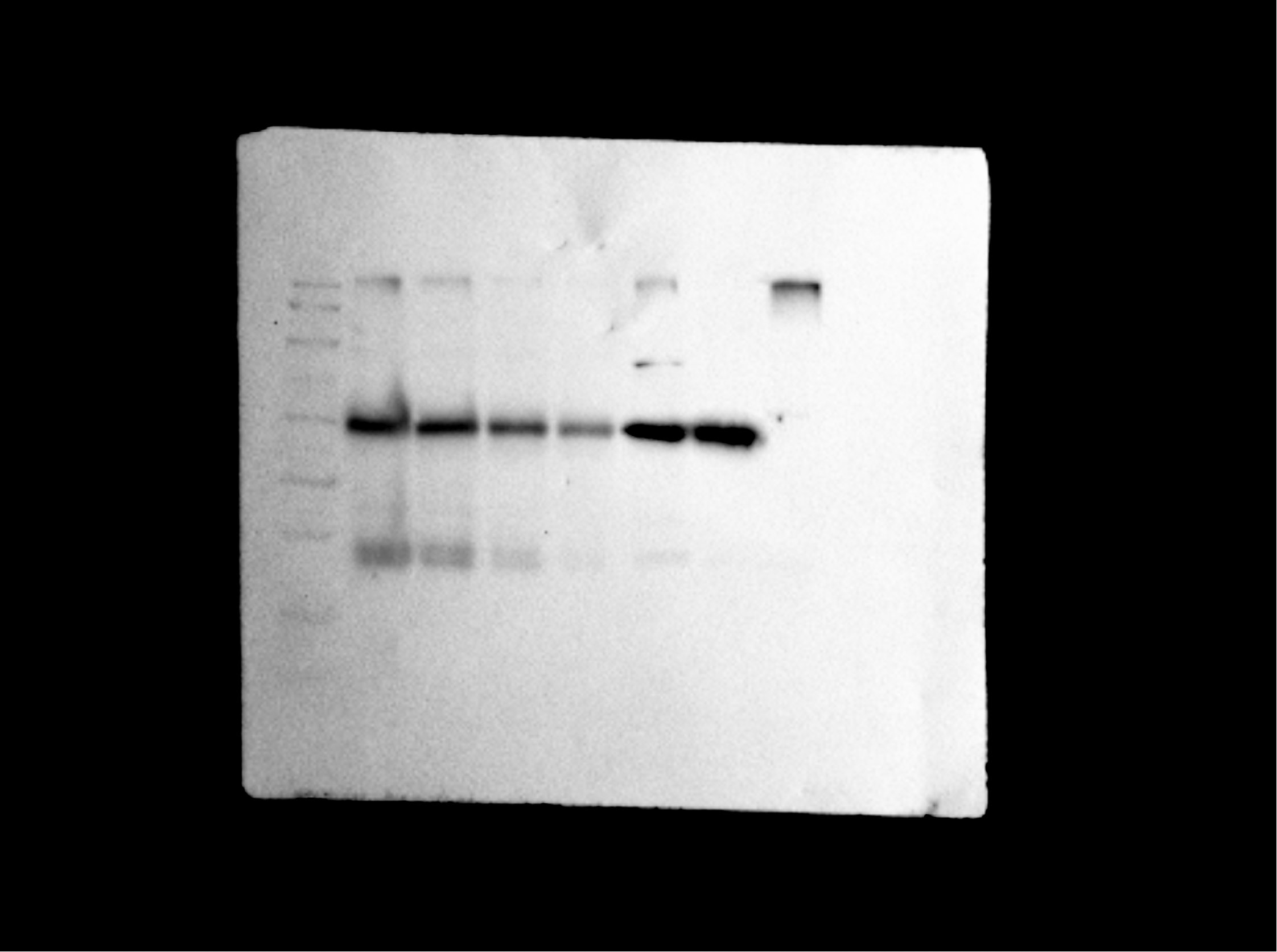


## Full and uncropped western blot of Figure S4I-1.jpg


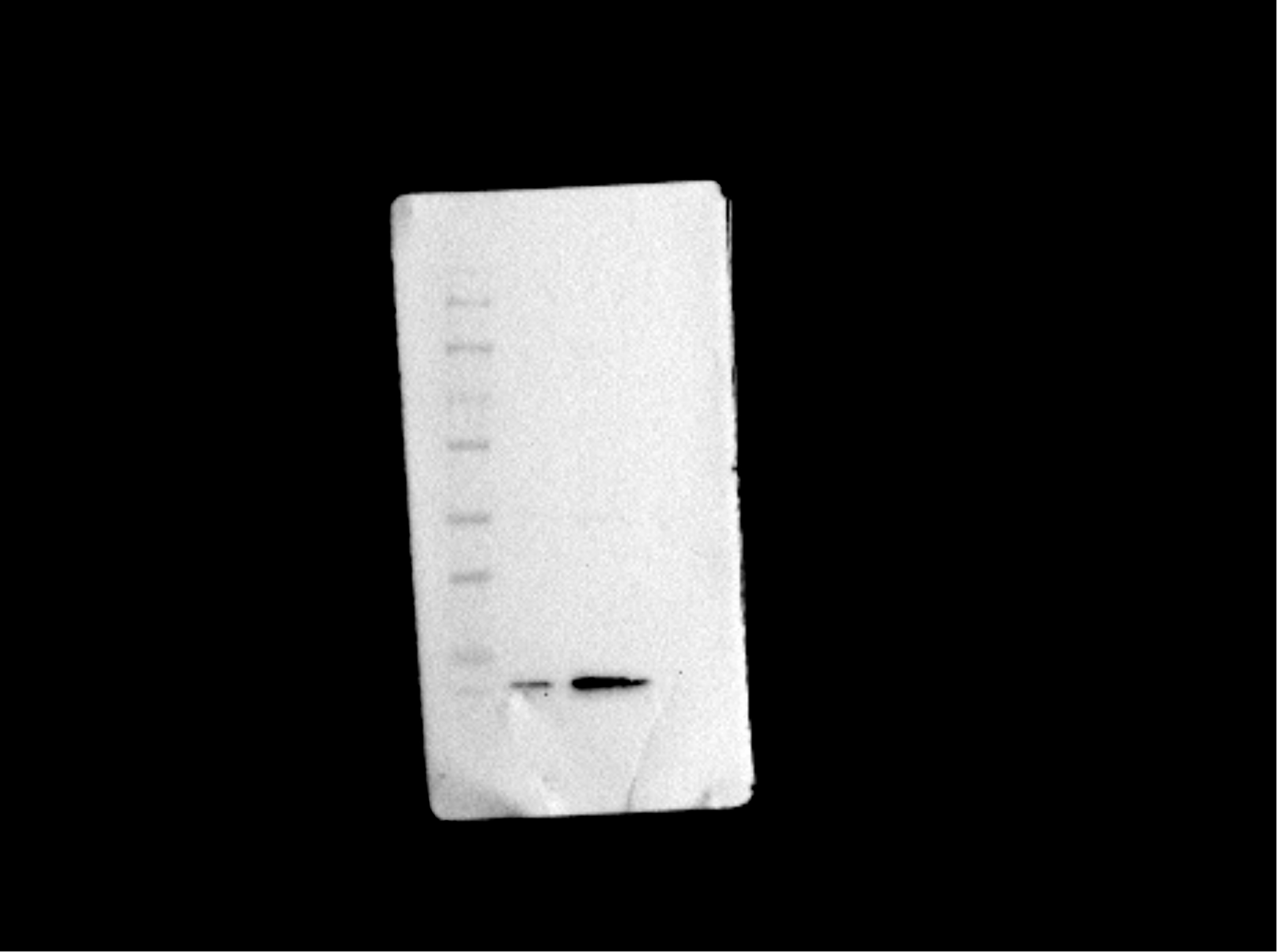


## Full and uncropped western blot of Figure S4I-2.jpg


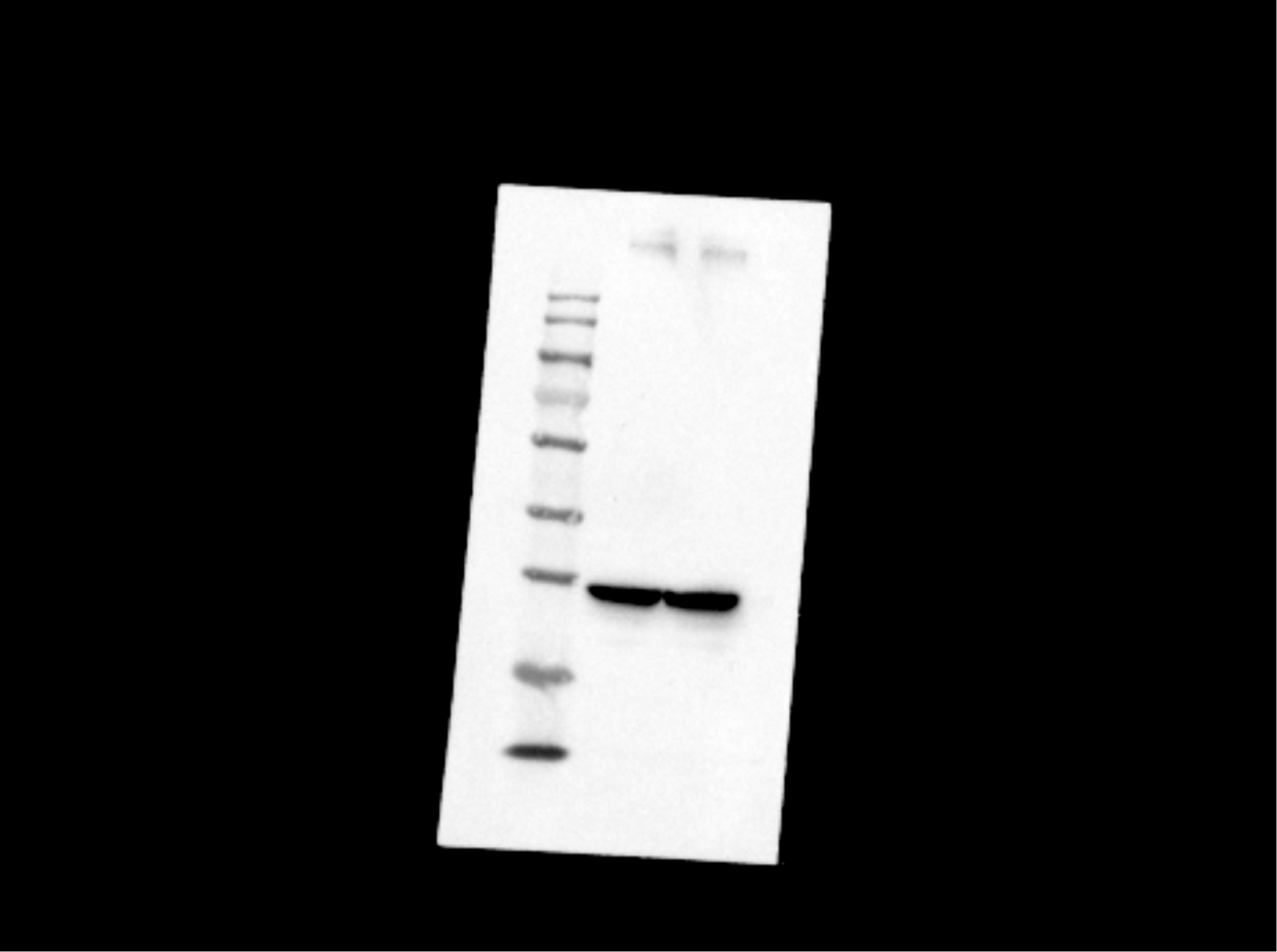


## Full and uncropped western blot of Figure S4J-1.jpg


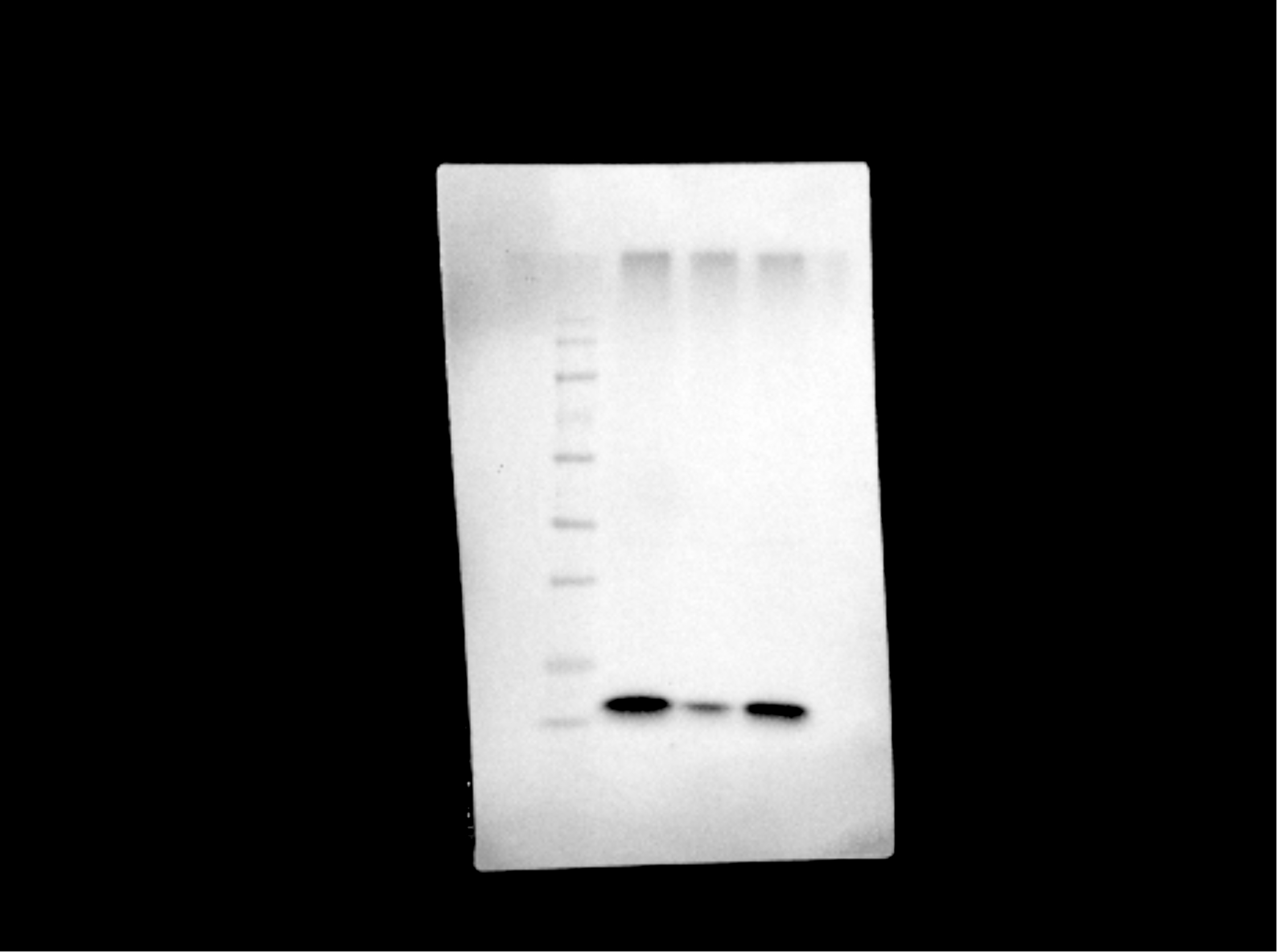


## Full and uncropped western blot of Figure S4J-2.jpg


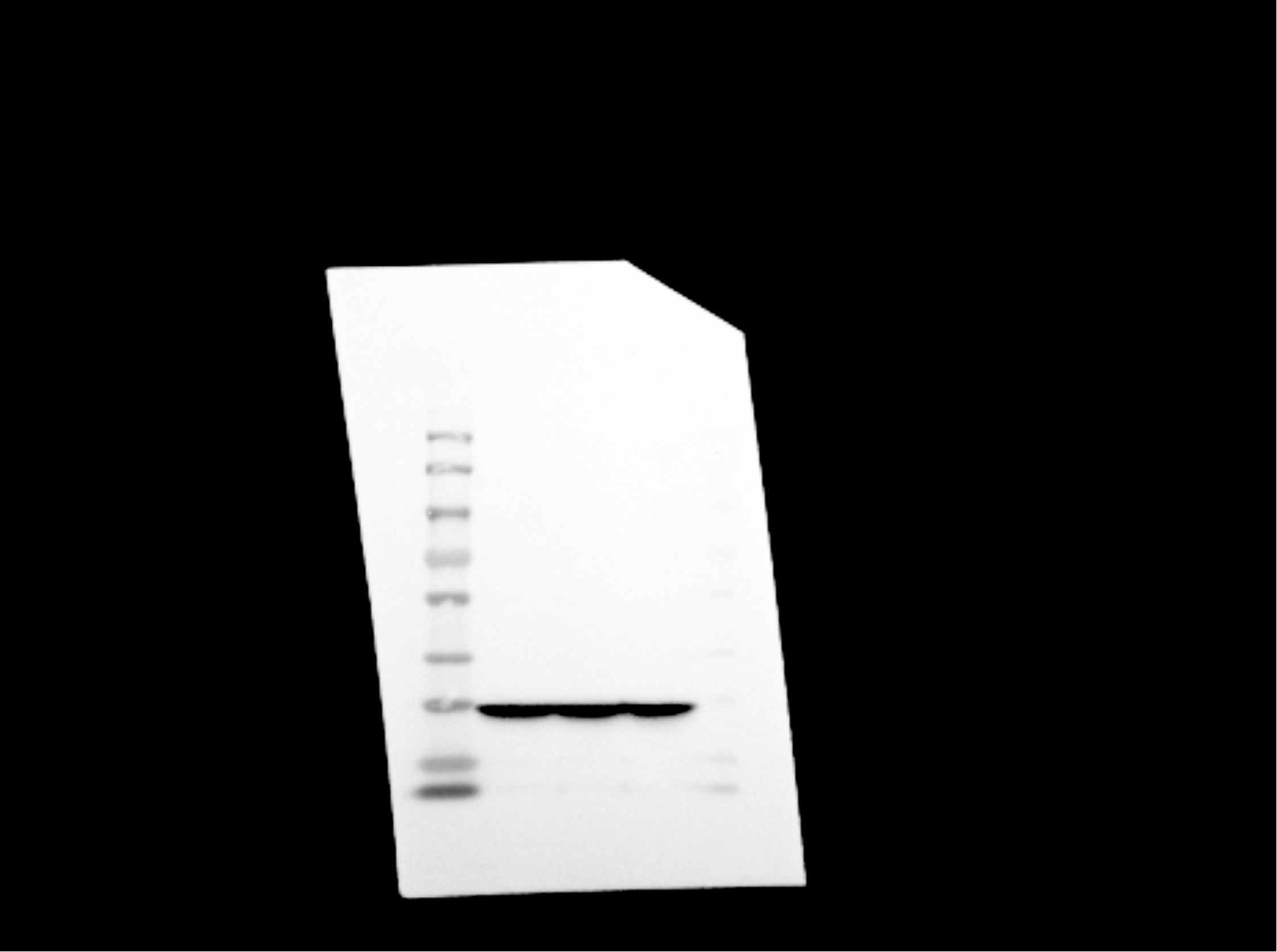

Supplement: Supplementary file 2 — Full and uncropped western blots [file 41420_2025_2690_MOESM2_ESM.docx]
